# Global decadal variability of plant carbon isotope discrimination and its link to gross primary production

**DOI:** 10.1111/gcb.15924

**Published:** 2021-10-18

**Authors:** Aliénor Lavergne, Deborah Hemming, Iain Colin Prentice, Rossella Guerrieri, Rebecca J. Oliver, Heather Graven

**Affiliations:** ^1^ Department of Physics Imperial College London London UK; ^2^ Met Office Hadley Centre Exeter UK; ^3^ Birmingham Institute of Forest Research Birmingham UK; ^4^ Department of Life Sciences Georgina Mace Centre for the Living Planet Imperial College London Ascot UK; ^5^ Grantham Institute – Climate Change and the Environment Imperial College London London UK; ^6^ Department of Biological Sciences Macquarie University North Ryde New South Wales Australia; ^7^ Department of Earth System Science Tsinghua University Beijing China; ^8^ Department of Agricultural and Food Sciences University of Bologna Bologna Italy; ^9^ UK Centre for Ecology and Hydrology Wallingford UK

**Keywords:** carbon isotope discrimination, forest ecosystems, gross primary production, JULES model, land carbon uptake, tree rings

## Abstract

Carbon isotope discrimination (Δ^13^C) in C_3_ woody plants is a key variable for the study of photosynthesis. Yet how Δ^13^C varies at decadal scales, and across regions, and how it is related to gross primary production (GPP), are still incompletely understood. Here we address these questions by implementing a new Δ^13^C modelling capability in the land‐surface model JULES incorporating both photorespiratory and mesophyll‐conductance fractionations. We test the ability of four leaf‐internal CO_2_ concentration models embedded in JULES to reproduce leaf and tree‐ring (TR) carbon isotopic data. We show that all the tested models tend to overestimate average Δ^13^C values, and to underestimate interannual variability in Δ^13^C. This is likely because they ignore the effects of soil water stress on stomatal behavior. Variations in post‐photosynthetic isotopic fractionations across species, sites and years, may also partly explain the discrepancies between predicted and TR‐derived Δ^13^C values. Nonetheless, the “least‐cost” (Prentice) model shows the lowest biases with the isotopic measurements, and lead to improved predictions of canopy‐level carbon and water fluxes. Overall, modelled Δ^13^C trends vary strongly between regions during the recent (1979–2016) historical period but stay nearly constant when averaged over the globe. Photorespiratory and mesophyll effects modulate the simulated global Δ^13^C trend by 0.0015 ± 0.005‰ and –0.0006 ± 0.001‰ ppm^−1^, respectively. These predictions contrast with previous findings based on atmospheric carbon isotope measurements. Predicted Δ^13^C and GPP tend to be negatively correlated in wet‐humid and cold regions, and in tropical African forests, but positively related elsewhere. The negative correlation between Δ^13^C and GPP is partly due to the strong dominant influences of temperature on GPP and vapor pressure deficit on Δ^13^C in those forests. Our results demonstrate that the combined analysis of Δ^13^C and GPP can help understand the drivers of photosynthesis changes in different climatic regions.

## Graphical abstract

This is the first study investigating decadal changes in carbon isotope discrimination (Δ^13^C) and documenting the links between Δ^13^C and gross primary production (GPP) across the globe using a novel data‐model approach. Modelled Δ^13^C trends vary strongly between regions but stay nearly constant when averaged over the globe during the recent historical period, which contrast with previous findings based on atmospheric carbon isotope measurements. Our results suggest that studying Δ^13^C and GPP together may inform about the most influential drivers of photosynthesis and thus help understanding temporal changes in the land carbon uptake.

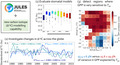

## 1 INTRODUCTION

Plant tissues have a ^13^C‐depleted isotopic signature relative to atmospheric CO_2_ because of the slower diffusion of heavier ^13^CO_2_ molecules through the stomata, and the preferential fixation of ^12^CO_2_ by the enzyme RuBisCO during carboxylation. This isotopic fractionation is well‐known as the discrimination against ^13^C, and denoted Δ^13^C (Park & Epstein, 1960). Despite decades of research, however, there is still incomplete knowledge of how and why Δ^13^C in C_3_ woody plants varies over decadal timescales, and across regions.

Studies investigating short‐term (seasonal to interannual) variations in Δ^13^C and their spatial patterns using stable carbon isotope ratios (δ^13^C) measured in C_3_ leaves have shown that changes in Δ^13^C depend on environmental conditions, including soil moisture and annual precipitation (Diefendorf et al., 2010; Kohn, 2010), atmospheric vapor pressure deficit (Lloyd & Farquhar, 1994; Wang, Prentice, Keenan, et al., 2017), temperature, atmospheric pressure (Cornwell et al., 2018), and thus elevation (Korner et al., 1988, 1991; Wang, Prentice, Davis, et al., 2017)—see Cernusak et al. (2013) for a review. Other studies using δ^13^C measured in tree rings (TRs) have suggested that Δ^13^C in C_3_ woody plants increases with rising atmospheric CO_2_ (Ehleringer & Cerling, 1995; Feng & Epstein, 1995; McCarroll et al., 2009; Treydte et al., 2001). Experimental studies investigating changes in Δ^13^C of (well‐watered) herbaceous C_3_ angiosperm plants growing at different CO_2_ levels (Schubert & Jahren, 2012, 2015) have also shown this pattern. In principle, Δ^13^C might increase with rising CO_2_ in mesic or well‐watered sites when stomata are fully open and thus the level of CO_2_ inside the leaf (*c*_i_) is high, reflecting the RuBisCO enzyme's preference for ^12^CO_2_ (Farquhar et al., 1982). However, even with rising CO_2_, Δ^13^C might decrease in dry regions when stomatal conductance is reduced and *c*_i_ is low (Farquhar et al., 1982).

The actual sensitivity of Δ^13^C to changes in CO_2_ continues to be debated. For example Sheldon et al. (2020) found no significant change in Δ^13^C values derived from a large global data set of leaf measurements from woody gymnosperms for the post‐1850 period. They suggested that fundamental differences in Δ^13^C responses to CO_2_ between plant functional groups might explain the apparent discrepancies between experimental and historical studies. Consistent with these findings, Hare and Lavergne (2021) used a model‐data fusion approach to show that the Δ^13^C in woody gymnosperms is less sensitive to CO_2_ than that in angiosperms. It has also been suggested that decadal‐scale plant physiological responses, including adjustments of stomatal size and density, could counteract the positive effect of CO_2_ on Δ^13^C values in woody C_3_ plants (Rayback et al., 2020; Saurer et al., 2004; Stein et al., 2020).

One study exploring ^13^CO_2_ budgets using atmospheric measurements and a box model (Keeling et al., 2017) estimated that plant Δ^13^C should have increased globally by 0.014 ± 0.007‰ ppm^–1^ over the period 1978–2014, in order to explain the magnitude of the decreasing trend observed in atmospheric δ^13^CO_2_ (attenuation of the Suess effect: Keeling, 1979). Suggested reasons for such an increase were the impact of CO_2_ on photorespiratory fractionation and (more importantly) mesophyll‐conductance fractionation. Their results imply that despite differing temporal variations in Δ^13^C among sites, Δ^13^C should have shown a global increase, when integrated over all ecosystems. However, the global Δ^13^C trend suggested by this study has not been independently validated.

In addition to the global Δ^13^C trend, the degree to which Δ^13^C can be used as proxy for interannual variations in gross primary production (GPP) is still unclear. During large‐scale drought, when continental‐scale net carbon uptake is low, so too is Δ^13^C, leading to a positive relationship between Δ^13^C and GPP (Peters et al., 2018; Randerson et al., 2002). This relationship is consistent with Belmecheri et al. (2014) findings in the (mesic) Harvard Forest using TR and eddy‐covariance (EC) flux measurements. However, other studies not only reported positive relationships between Δ^13^C and TR growth in relatively dry regions, but also negative ones in wet and/or cold regions (del Castillo et al., 2014; Shestakova et al., 2019; Voelker et al., 2014). Because TR growth is an indicator of GPP (Babst et al., 2014), their results suggest that even the sign of the GPP‐Δ^13^C relationship could vary with environment.

Here we explore mean values and trends in global Δ^13^C of C_3_ woody plants over the 1979–2016 period using the Joint UK Land Environment Simulator (JULES) model (version 5.6), newly equipped with a carbon isotopic modelling capability. We first evaluate the model at the local scale, using a large network of δ^13^C data from leaves and TRs to assess which *c*_i_ model in JULES shows the best agreement with observations. We then analyze the predicted Δ^13^C values and trends from the global historical simulations and quantify the relative contributions to the global trend. We also investigate the sign, magnitude, and drivers of the relationship between Δ^13^C and GPP across regions. We aim to answer three questions: (1) Is the global increase in plant Δ^13^C suggested by Keeling et al. (2017) reproduced by JULES? (2) What are the contributions from photorespiratory and mesophyll effects to the global Δ^13^C trend? and (3) Is the correlation between Δ^13^C and GPP driven by environmental conditions, as suggested by TR studies?

## 2 MATERIALS AND METHODS

### 2.1 Description of the JULES model

JULES is the land surface component of the UK Earth System Model (Sellar et al., 2019) simulating simultaneously the fluxes of carbon, water and energy between the land surface and the atmosphere (https://jules.jchmr.org). Vegetation in the model is represented as plant functional types (PFTs), which differ in their biochemical and biophysical properties. A full description of the model is available in previous publications (Best et al., 2011; Clark et al., 2011; Harper et al., 2016).

#### 2.1.1 Leaf‐level photosynthesis and soil water stress factor

The potential assimilation rate (*A*_p_, μmol m^−2^ s^−1^) for C_3_ plants is predicted using the standard Farquhar et al. (1980) model as the minimum of two limiting factors (electron transport *A*_J_ and carboxylation rate *A*_C_, see Text S1) minus the dark mitochondrial respiration (*R*_d_; μmol m^−2^ s^−1^). *A*_C_ is determined by the maximum carboxylation rate (*V*_cmax_, μmol m^−2^ s^−1^), which regulates the RuBisCO enzymatic capacity for carbon fixation, and *A*_J_ depends on light (with a maximum value determined by the maximum electron‐transport rate, *J*_max_, μmol m^−2^ s^−1^). Both limiting factors also depend on the leaf intercellular partial pressure of CO_2_ (*c*_i_, Pa) and on the CO_2_ compensation point in the absence of *R*_d_ (Γ*, Pa). *R*_d_ is proportional to *V*_cmax_ but varies among PFTs through the parameter *f*_d_ (Harper et al., 2016). The net assimilation rate (*A*_n_) resulting from biochemical limitations of photosynthesis due to soil water stress is then estimated as:

(1) $$A_{n}=\left(W‐R_{d}\right)\beta_{\text{soil}},$$

*W* is the smoothed minimum of *A*_C_ and *A*_J_. *β*_soil_ is a dimensionless soil water stress factor (*β*_soil_) ranging between 1 (well‐watered plants) and 0 (no available water) and determined based on the soil characteristics (texture and type) following the Van Genuchten (1980) model.

*β* (unitless) is calculated in each grid box for each soil layer *k* as the function of volumetric soil water content in each layer of the root zone ($\theta_{k}$, m^3^ m^−3^) using:

(2a) $$\beta_{k}=\left\{\begin{array}{cc} 1 & \theta_{k}>\theta_{\text{upp},k}\\ \frac{\theta_{k}‐\theta_{w,k}}{\theta_{\text{upp},k}‐\theta_{w,k}} & \theta_{w,k}<\theta_{k}\leq \theta_{\text{upp},k}\\ 0 & \theta_{k}\leq \theta_{w,k} \end{array}\right.,$$

with

(2b) $$\theta_{\text{upp},k}=\theta_{w,k}+\left(1‐p_{0}\right)\left(\theta_{c,k}‐\theta_{w,k}\right).$$

$\theta_{\text{c,k}}$ is the critical θ above which plants are unaffected by water stress (defined as the field capacity, m^3^ m^−3^) and $\theta_{w,k}$ is the wilting θ below which water stress is at its maximum (or permanent wilting point, m^3^ m^−3^) in each soil layer *k*. $\theta_{\text{upp},k}$ is the water content at which plants start to become water stressed in each soil layer *k*. *p*_0_ is a parameter governing the threshold at which the plant starts to experience water stress due to lack of water in the soil (ranging between 0 and 1). A *p*_0_ value equal to 0.4 is used here to delay the onset of soil water stress, consistently with recent studies (Harper et al., 2021; Williams et al., 2019). The overall *β*_soil_ is calculated based on the water stress in each layer estimated from Equation ((2a), (2b)) and the fraction of root mass in each soil layer *k* (*r*_k_):

(3) $$\beta_{\text{soil}}=\sum_{k}^{n_{\text{soil}}}r_{k}\beta_{k},$$

where *n*_soil_ is the number of soil layers (equal to 14 in this study).

#### 2.1.2 Leaf intercellular CO_2_ models tested in JULES

The leaf‐level stomatal conductance for carbon (*g*_sc_; mol m^−2^ s^−1^) is related to *A*_n_ from Equation (1) via the CO_2_ diffusion equation as:

(4) $$g_{\text{sc}}=\frac{A_{n}}{\left(c_{a}‐c_{i}\right)},$$

where *c*_a_ and *c*_i_ are the ambient and leaf‐intercellular CO_2_ (in μmol mol^−1^ here), respectively.

Two representations of *c*_i_ have been incorporated into JULES based on the Jacobs (1994) and Medlyn et al. (2011) stomatal conductance models. The Jacobs (1994) model originally implemented into JULES (Best et al., 2011) relates *c*_i_/*c*_a_ to leaf humidity deficit as:

(5) $$c_{i}=\left(c_{a}‐\Gamma^{*}\right)f_{0}\left(1‐\frac{d_{q}}{d_{q_{\text{crit}}}}\right)+\Gamma^{*}.$$

*d*_q_ and $d_{q_{\text{crit}}}$ are the specific and critical humidity deficit at the leaf surface (kg kg^−1^). *f*_0_ is the PFT‐dependent *c*_i_/*c*_a_ at the leaf‐specific humidity deficit.

The Medlyn et al. (2011) model incorporated and validated in JULES version 5.5 (Oliver et al., 2018) assumed that stomatal aperture is regulated to maximize carbon gain while simultaneously minimizing water loss so that:

(6) $$c_{i}=\frac{c_{a}g_{1}}{g_{1}+\sqrt{D}}.$$

*D* (kPa) is the leaf‐to‐air vapor pressure deficit and *g*_1_ (kPa^0.5^) is a PFT‐dependent fitted parameter representing the sensitivity of *g*_sc_ to *A*, that is, the normalized water use efficiency.

Two other stomatal models have been incorporated into JULES version 5.6 for this study, the Leuning (1995) and the Prentice et al. (2014) models. The Leuning (1995) model is a modified version of the simple Ball et al. (1987) model and is widely used in land surface modelling:

(7) $$c_{i}=c_{a}‐\frac{1}{g_{0}+a_{1}{\left(1+\frac{D}{D_{0}}\right)}^{‐1}/\left(c_{a}‐\Gamma^{*}\right)}.$$

*g*_0_ (μmol m^−2^ s^−1^), *D*_0_ (kPa), *a*_1_ (unitless) are empirically fitted parameters representing the residual stomatal conductance and the sensitivity of stomata to changes in *D* and *A*, respectively.

The Prentice et al. (2014) model is based on the least‐cost optimality hypothesis which assumes that leaves minimize the summed unit costs of transpiration and carboxylation so that:

(8a) $$c_{i}=\left(c_{a}‐\Gamma^{*}\right)\frac{\xi }{\xi +\sqrt{D}}+\Gamma^{*},$$

where

(8b) $$\xi =\sqrt{\beta \frac{\left(K+\Gamma^{*}\right)}{1.6\eta^{*}}}.$$

*β* (unitless) is the ratio of cost factors for carboxylation and transpiration at 25°C, which may vary with changes in plant‐available soil water (Lavergne et al., 2020), but is set constant here because no mechanistic formulation for the soil water stress has been proposed yet. *K* is the effective Michaelis constant for Rubisco‐limited photosynthesis at a given partial pressure of O_2_ (Pa). η* (unitless) is the dynamic viscosity of water relative to its value at 25°C, assumed constant here (equal to 1). Both Γ* and *K* are calculated from their respective values at 25°C and 99.1 kPa (and activation energy) following Bernacchi et al. (2001).

All these models (Equations (5), (6), (7), (8a), (8b), (9a), (9b), (10), (11), (12), (13), (14), (15a), (15b), (15c), (16), (17)) implicitly assume infinite mesophyll conductance (*g*_m_) and therefore that the ratio of chloroplastic (*c*_c_) to ambient (*c*_a_) CO_2_ (*c*_c_/*c*_a_) is equal to *c*_i_/*c*_a_. This is an oversimplification as *g*_m_ is low enough to cause a drawdown from *c*_i_ to *c*_c_ (Flexas et al., 2012), leading to lower *c*_c_/*c*_a_ compared to *c*_i_/*c*_a_. An alternative formulation for the Prentice model assuming finite *g*_m_ has been proposed by Wang, Prentice, Keenan, et al. (2017) and is also tested here:

(9a) $$c_{c}=\left(c_{a}‐\Gamma_{c}^{*}\right)\frac{\xi_{c}}{\xi_{c}+\sqrt{D}}+\Gamma_{c}^{*},$$

where

(9b) $$\xi_{c}=\sqrt{\beta_{c}\frac{\left(K_{c}+\Gamma_{c}^{*}\right)}{1.6\eta^{*}\left(1+g_{\text{sc}}/g_{m}\right)}}.$$

*β* (unitless) is equivalent to *β*_c_ assuming finite *g*_m_. $\Gamma_{c}^{*}$ and *K*_c_ are equivalent to Γ* and *K* assuming finite *g*_m_ calculated following Bernacchi et al. (2002). The *g*_sc_/*g*_m_ ratio is assumed constant here.

#### 2.1.3 Upscaled canopy fluxes of carbon and water

The leaf‐level *A*_n_, *R*_d_ and *g*_sc_ in each layer of the canopy (i) are scaled by the fraction of leaf area index (LAI, m^2^ m^−2^) for sunlit and shaded leaves and summed over all model layers (*n*, equal to 10 here) to obtain canopy‐scale estimates *A*_can_, *R*_dc_ and *G*_sc_ as:

(10) $$X_{c}=\sum_{i=1}^{n}\left[\left(f_{\text{sun},i}X_{\text{sun},i}+\left(1‐f_{\text{sun},i}\right)X_{\text{shade},i}\right)\cdot \frac{\text{LAI}}{n}\right],$$

where *X* is the variable considered and, *f*_sun,i_ and *f*_shade,i_ are the fractions of sunlit and shaded leaves for each canopy layer *i*, respectively.

The GPP (gC m^−2^ s^−1^) is then estimated as:

(11) $$\text{GPP}=0.012\left(A_{\text{can}}+R_{\text{dc}}\beta_{\text{soil}}\right).$$

The factor 0.012 converts from mol CO_2_ m^−2^ s^−1^ to kg C m^−2^ s^−1^.

The surface latent heat flux (LE, W m^−2^) is calculated using the humidity gradient between the surface and atmospheric reference height *z*_1_ following Cox et al. (1999):

(12) $$\text{LE}=\psi \frac{L\rho }{r_{a}}\left(q_{\text{sat}}\left(T_{s}\right)‐q_{1}\right).$$

ψ is the factor determined from the proportions of canopy evaporation, bare soil evaporation, transpiration by vegetation through the stomata, and sublimation from snow. *L* is the latent heat of vaporization of water (J kg^−1^), ρ is the surface air density (kg m^−3^), *r*_a_ is the aerodynamic resistance (s m^−1^), *q*_sat_ (*T*_s_) is the saturated specific humidity at surface temperature *T*_s_ (kg kg^−1^), and *q*_1_ is the specific humidity at reference height *z*_1_ (kg kg^−1^). LE is converted from energy (W m^−2^) into mass (evapotranspiration, ET in kgH_2_O m^−2^ s^−1^) by dividing LE with *L*.

The transpiration flux (*T*_r_, kgH_2_O m^−2^ s^−1^) is calculated for the vegetated fraction of the grid box *f*_v_ (unitless) as:

(13) $$T_{r}=\left(1‐f_{a}\right)f_{v}\frac{\rho }{r_{a}+r_{c}}\left(q_{\text{sat}}\left(T_{s}\right)‐q_{1}\right).$$

*f*_a_ is the wet fraction of the canopy (unitless) and *r*_c_ is the canopy resistance (s m^−1^) inversely related to the canopy *g*_sw_ (*G*_sw_, m s^−1^). *G*_sw_ is related to *G*_sc_ following Equation (4) as:

(14) $$G_{\text{sw}}=\frac{1}{r_{c}}=1.6{\text{RT}}_{s}\frac{A_{\text{can}}}{c_{a}‐c_{i}}=1.6{\text{RT}}_{s}G_{\text{sc}},$$

with *R* the ideal gas constant (J K^−1^ mol^−1^).

#### 2.1.4 Implementation of stable carbon isotopes into JULES

Assuming infinite boundary layer conductance and negligible fractionation during mitochondrial respiration (Evans & von Caemmerer, 2013), Δ^13^C in C_3_ plants depends on the isotope fractionations due to CO_2_ diffusion across the stomata pore (*a* = 4.4‰) and the mesophyll cell (*a*_m_ = 1.8‰), and due to effective RuBisCO carboxylation (*b* = 30‰) and photorespiration (*f* = 12 ± 4‰) following Farquhar et al. (1982):

(15a) $$\Delta {\;}^{13}C_{c}=a\frac{c_{a}‐c_{i}}{c_{a}}+b\frac{c_{c}}{c_{a}}‐f\frac{\Gamma_{c}^{*}}{c_{a}}+a_{m}\frac{c_{i}‐c_{c}}{c_{a}}.$$

Assuming infinite *g*_m_, Equation (15a) can be simplified as:

(15b) $$\Delta {\;}^{13}C=a+\left(\bar{b}‐a\right)\frac{c_{i}}{c_{a}}‐f\frac{\Gamma^{*}}{c_{a}},$$

where $\bar{b}$ = 28‰ following Ubierna and Farquhar (2014) to account for the missing mesophyll term. Finally, assuming no fractionation during photorespiration, Equation (15b) is simplified:

(15c) $$\Delta {\;}^{13}C=a+\left(b^{\prime }‐a\right)\frac{c_{i}}{c_{a}},$$

where *b*′ = 27‰ following Farquhar et al. (1982) to account for the missing mesophyll and photorespiration terms. We incorporated these three variants of the discrimination model into JULES version 5.6. Note that for Equation (15a), only the Prentice stomatal model (Equation (9a), (9b)) was used because the other three models do not predict *c*_c_.

### 2.2 Model configurations, simulations, and evaluation with observations

#### 2.2.1 Model setup: Configurations and environmental drivers

We ran the model on the NERC JASMIN platform (http://www.jasmin.ac.uk/) using the Rose/Cylc suite control system. We used the suite control setup to initialize, configure, spin up, and run the historical simulations. We used a JULES configuration (Rose suite u‐bx886) with 13 surface tiles consisting of nine PFTs (tropical broadleaf evergreen trees, temperate broadleaf evergreen trees, broadleaf deciduous trees, needle‐leaf evergreen trees, needle‐leaf deciduous trees, C_3_ grasses, C_4_ grasses, evergreen shrubs, and deciduous shrubs) following Harper et al. (2016) and four non‐vegetated surface types (urban, inland water, bare soil, and ice). The PFT‐dependent parameters values of the photosynthesis and *c*_i_ models tested here for the five forest PFTs are reported in Table S1.

The model was forced by the WFDEI meteorological reanalysis data set at 0.5° × 0.5° spatial and 3 h temporal resolution over 1979–2016 (Weedon et al., 2014). All forcing variables were interpolated down to half‐hourly resolution. We ran JULES with prescribed annual mean atmospheric CO_2_ concentrations from NOAA ESRL (www.esrl.noaa.gov/gmd/ccgg/trends/). We used a fixed land cover mask based on the European Space Agency's Land Cover Climate Change Initiative global vegetation distribution (Poulter et al., 2015) and prescribed the soil properties using ancillary data based on the Van Genuchten (1980) model (Slevin, 2017). The model was spun up for a maximum of three cycles of 38 years length. The spin‐up simulations were performed starting with existing climatology provided by the meteorological data as initial condition, by iterative repeating the years of simulation (1979–2016) until the solutions converge on two in‐soil prognostic variables following these criteria: (1) for soil moisture: to within 1% of content, (2) for soil temperature: to within 0.1°K.

#### 2.2.2 Model simulations at the point‐scale and evaluation with observations

We first evaluated the JULES model regarding its representation of the controls by leaf stomata of the coupled carbon and water cycles to determine which *c*_i_ model should be used for the global simulations. We tested different combinations of the discrimination and stomatal models described above (see Table S2) running the model at the point‐scale where isotopic data for C_3_ woody plants were available and compared the resulting Δ^13^C predictions with the observational data set described below.

##### Simulations at sites with leaf or TR isotopic measurements

We used the δ^13^C data set used in Lavergne, Sandoval, et al. (2020) (referred to as ‘global network’, Figure S1a), which comprises leaf δ^13^C data from Diefendorf et al. (2010), Cornwell et al. (2018) and Sheldon et al. (2020) and TR δ^13^C data from Lavergne, Voelker, et al. (2020). We additionally used TR δ^13^C data derived from other recent compilations (Adams et al., 2020; Belmecheri et al., 2021; Mathias & Thomas, 2021). From an original compilation of 545 TR chronologies spanning the 1901–2016 period, we only selected those with at least 25 years of measurements over the 1979–2012 period (total of 151 chronologies). We reduced the analysis period to 1979–2012 (not 1979–2016) because only a few TR chronologies were available after 2012.

We then calculated ∆^13^C as:

(16) $$\Delta {\;}^{13}C=\frac{\delta^{13}{\text{CO}}_{2}‐\left(\delta^{13}C_{\text{PM}}‐f_{\text{post}}\right)}{1+\left(\delta^{13}C_{\text{PM}}‐f_{\text{post}}\right)/1000},$$

where δ^13^CO_2_ and δ^13^C_PM_ (‰) are the stable isotopic compositions of the atmospheric CO_2_ and of the plant material considered (bulk leaf or α‐cellulose in TR), respectively. Yearly δ^13^CO_2_ values were derived from Graven et al. (2017). *f*_post_ quantifies the sum of fractionations beyond those associated with the production of the primary photosynthetic assimilates—δ^13^C being higher in wood than in leaves (Cernusak et al., 2009). Because the leaf and TR data from the global network were not available at the same sites and, thus, the post‐photosynthetic fractionations could not be estimated for each TR site, we used a standard estimate (*f*_post_ = 2.1 ± 1.2‰ between bulk leaf and α‐cellulose in TR; Belmecheri and Lavergne, 2020) assuming that the resulting ∆^13^C was equivalent to bulk leaf‐derived ∆^13^C. Thus, we considered that the fractionation effects beyond those occurring within the leaf were constant for all tree species and sites, and across years.

We then compared the leaf and TR‐derived Δ^13^C data with the predicted Δ^13^C values from the different combinations of the discrimination and stomatal models. We averaged the predicted Δ^13^C values over summer months (i.e., June–August for Northern Hemisphere and December–February for Southern Hemisphere). Alternatively, we tested weighting the predicted Δ^13^C for the PFT considered using the predicted GPP values for each year as:

(17) $$\Delta {\;}^{13}C_{w}=\sum_{t}\frac{\Delta {\;}^{13}C_{t,\text{PFT}}\times {\text{GPP}}_{t,\text{PFT}}}{\sum_{t}{\text{GPP}}_{t,\text{PFT}}}.$$

For comparison of predicted values with the leaf‐derived Δ^13^C data set, we used Taylor diagrams (Taylor, 2001) to provide a statistical summary of the predictive skills of the model for the different simulations to reproduce the observational data set in terms of their Pearson's correlation coefficient (*r*), normalized standard deviation (SD) and centered root‐mean‐square error (RMSE). For comparison with the TR‐derived Δ^13^C timeseries, we estimated the Theil‐Sen's slope and interannual variability (IAV) of each model prediction and TR timeseries and compared the resulting values between each other at each site. The IAV was calculated as the SD of the difference between the Δ^13^C values and the Theil‐Sen's slope (i.e., the median of the slopes of all lines through pair of points; Sen, 1968).

##### Simulations at EC flux stations

We ran the model at EC flux stations where both carbon and water flux measurements and δ^13^C data in different parts of the plants were measured simultaneously for several years (referred to as ‘EC flux stations’, Figure S1b). The simulations at these sites allowed us to test the impact of the stomatal model selected on the predicted canopy fluxes. Using leaf and TR δ^13^C data at these sites we can also quantify the post‐photosynthetic fractionations and compare them to the standard value we used for the other TR sites.

We used the isotopic data set from Hemming et al. (2005) providing δ^13^C measurements in bulk leaf and stem at nine EC stations from the EU CarboEuropeFlux project in 2001 and/or 2002. We also exploited the published δ^13^C data set in bulk leaves for a few individual years (Guerrieri et al., 2016) and α‐cellulose in TR for the 1982–2012 period (Guerrieri et al., 2019) at eight AmeriFlux stations (see Table S3). We then estimated the apparent *f*_post_ for each site and each year as the difference between the leaf and wood δ^13^C values. For the Hemming et al. (2005) data set, we corrected the obtained *f*_post_ values by 1.1‰ to account for differences between bulk and α‐cellulose TR δ^13^C following Guerrieri et al. (2017) estimates.

Half‐hourly or hourly GPP and LE data were extracted from the FLUXNET‐2015 Tiers 1 product (GPP_NT_VUF_REF and LE_F_MDS, respectively) when available for sites studied for the CarboEuropeFlux (BE‐Bra, DE‐Hai, DE‐Tha, DK‐Sor FI‐Hyy, FR‐LBr, IT‐Col, It‐Lav, and NL‐Loo) and AmeriFlux (US‐MMS and US‐Ha1) stations (Table S3). We screened and filtered the data as follows: (1) we used data flagged as good, (2) we removed data errors (*D* < 0.01 kPa), (3) we removed nighttime data using the nighttime/daytime flag based on potential radiation when provided or using the photosynthetic photon flux density (PPFD) variable (PPFD >0 during daytime), and (4) we excluded non transpirational water fluxes by removing days with precipitation and the subsequent 24 h. We converted GPP into gC m^−2^ day^−1^ and estimated ET (in kg H_2_O m^−2^ day^−1^) from LE and air temperature. We then aggregated GPP and ET into means over the growing season (May–September) and compared the resulting observations with the simulations for the same period using Taylor diagrams.

#### 2.2.3 Global simulations over 1979–2016

We ran the model at the global scale over the 1979–2016 period using the best stomatal model found with the point‐scale simulations (referred to as ‘global run’) and analyzed global mean values and trends in Δ^13^C predicted by JULES for the different versions of the discrimination model. For this global run, we assumed an isotopic fractionation due to Rubisco carboxylation of 30‰ for all discrimination models (Equations (15a), (15b), (15c)) to allow a fair intercomparison of the simulations. The predicted Δ^13^C for the five forest PFTs were weighted for each PFT using the GPP values and then averaged as in Equation (17) to produce one weighted‐Δ^13^C value per year and per grid‐point. In the following we use the term Δ^13^C when referring to weighted‐Δ^13^C. To explore the environmental drivers of Δ^13^C trends across the globe, we grouped the grid‐points for ranges of 5°C in annual air temperature (*T*_air_), 0.4 kPa in vapor pressure deficit (*D*) and 0.1 in soil water availability (*β*_soil_) and calculated the average of the Δ^13^C trends within each group (referred to ‘trend score’). We used a similar approach for exploring patterns of GPP trends across the globe.

To quantify the relative contributions of photorespiratory and mesophyll effects to the global Δ^13^C trend, we compared the simulation runs for the different discrimination models. To determine the sign and magnitude of the Δ^13^C‐GPP relationship across the globe and their links to environmental conditions, we calculated the Pearson's *r* between simulated annually averaged Δ^13^C and GPP at each grid point. We then grouped the resulting Δ^13^C‐GPP grid‐point correlations for the different ranges of annual *T*_air_, *D* and *β*_soil_ values and calculated the average of the Pearson's *r* within each group (referred to ‘correlation score’). Finally, to investigate the relative environmental dependencies of Δ^13^C and GPP within each correlation score group, we performed multiple regression models of Δ^13^C and GPP against their common drivers (i.e., *T*_air_, *D* and atmospheric CO_2_) for the different groups.

## 3 RESULTS

### 3.1 Selection of the best configuration of JULES using a data‐model approach

#### 3.1.1 Predicted mean Δ^13^C values: Comparisons with leaf isotopic measurements

**FIGURE 1:**
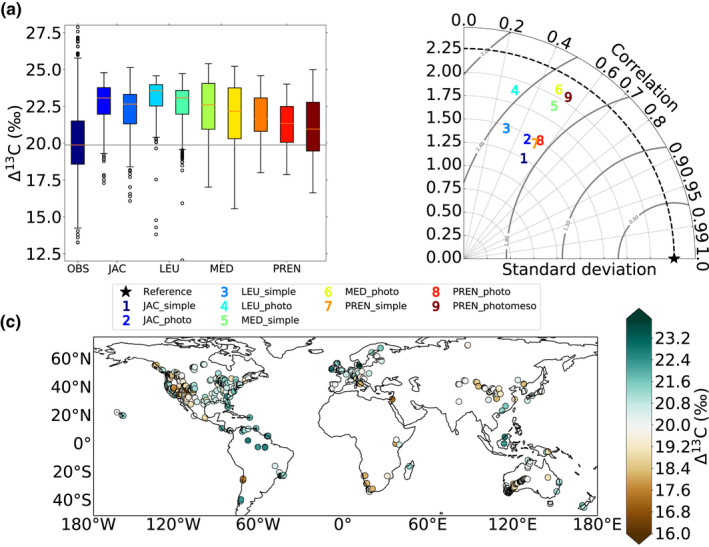
Impacts of stomatal and discrimination representations in JULES on the predicted Δ^13^C values as compared to the global network of leaf Δ^13^C measurements. Boxplots (a) and Taylor diagram (b). Leaf‐derived Δ^13^C values across the globe (c)

The observed leaf‐derived Δ^13^C averaged 20.1 ± 2.3‰ (mean ± SD; *n* = 1558; Figure 1a) and was higher in tropical areas, northern Europe and the east coast of northern America, but lower in mountainous regions, mainland Australia and the west coast of northern America (Figure 1c). All Δ^13^C predictions derived from the four stomatal models tested in JULES tended to overestimate median Δ^13^C and underestimate the SD in Δ^13^C as observed in the leaf isotopic data set (Figure 1a,b). Using the simple version of the discrimination model, the Δ^13^C values were overestimated by 2.8 ± 0.8‰ but incorporating the photorespiratory effect reduced the difference by 0.4‰ (2.4 ± 0.7‰; Figure 1a). The incorporation of the mesophyll effect in the Prentice model resulted in a further reduction of the bias, leading to a difference of around 1.1‰ between observed and predicted Δ^13^C median values. Overall, using the Prentice model combined with the discrimination model incorporating both photorespiratory and mesophyll effects produced the highest Pearson's *r* and the lowest RMSE (Table 1; Figure 1b).

**TABLE 1:** Pearson's correlation coefficients (*r*) between leaf‐derived and predicted Δ^13^C values for each stomatal and discrimination model. All correlations are significant at *p* < .001. Summer months (i.e., June–August for Northern Hemisphere and December–February for Southern Hemisphere)

Stomatal model	Discrimination model	Summer months	Weighted by GPP
Jacobs	Simple	0.51	0.36
	Photorespiration	0.46	0.29
Leuning	Simple	0.31	0.36
	Photorespiration	0.29	0.27
Medlyn	Simple	0.51	0.28
	Photorespiration	0.49	0.24
Prentice	Simple	0.52	0.43
	Photorespiration	0.54	0.42
	Photorespiration + finite mesophyll	0.54	0.44

#### 3.1.2 Predicted Δ^13^C trends and IAV: Comparisons with tree‐ring isotopic timeseries

**FIGURE 2:**
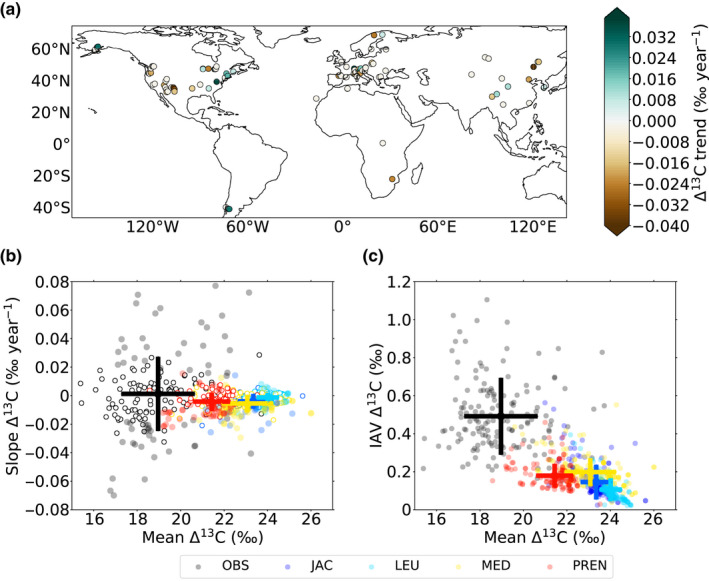
Tree‐ring‐derived Δ^13^C trends across the globe over the 1979–2012 period (a). Theil‐Sen's slopes (b) and interannual variations (c) against mean Δ^13^C values derived from the tree‐ring isotopic measurements and the (summer average) JULES predictions for each of the stomatal models, that is, Jacobs (blue), Leuning (light blue), Medlyn (yellow), and Prentice (red). In (b), the empty and full dots correspond to nonsignificant (*p* > .05) and significant (*p* < .05) slopes, respectively

Overall, TR Δ^13^C stayed relatively constant when considering all sites together (*p* = .097), but we found different patterns of variations when looking at the individual TR timeseries. While Δ^13^C in 95 out of 151 sites (63%) stayed nearly constant over 1979–2012, 29 sites showed a significant increasing trend (average 0.034 ± 0.006‰ year^−1^) and 27 depicted a negative trend (−0.037 ± 0.07‰ year^−1^). The sites with positive trends were mainly located in the east coast of northern America, whereas those with decreasing were situated in the west coast of northern America and eastern Asia (Figure 2a). All stomatal models overestimated mean Δ^13^C values by 1.8‰ to up to 5.0‰ compared to the global TR compilation (Figure 2b), which was higher than the bias reported with the leaf isotopic data set. The amplitude of the trends and the IAV of Δ^13^C predicted by JULES were lower than those of the observational network (SDs of slopes of 0.02 vs. 0.08‰ year^−1^, and average IAV of 0.2 ± 0.1‰ vs. 0.5 ± 0.2‰, respectively; Figure 2b,c). It is possible that the uncertainty or variability in *f*_post_ increased the bias between the TR‐derived ∆^13^C and the JULES predictions.

Using the published δ^13^C data set in bulk leaves and corresponding α‐cellulose TR at the AmeriFlux stations to estimate the post‐photosynthetic fractionation, we found large variations in *f*_post_ not only across species and sites, but also between the 2 years of available data, with values averaging 4.1 ± 1.1‰ (Table 2). This estimate falls within the higher range of the standard *f*_post_ values reported in the literature (i.e., 2.1 ± 1.2‰). Using the bulk leaf and stem isotopic data set from the CarboEuropeFlux stations corrected by 1.1‰ to account for difference between bulk and α‐cellulose stem (Guerrieri et al., 2017), we found an apparent *f*_post_ of around 2.2 ± 1.0‰ (Table 2). While this last estimate is consistent with the standard *f*_post_ values we used in the ‘global network’ analyses, accounting for the *f*_post_ calculated at the AmeriFlux stations would result in lower TR‐derived ∆^13^C than previously estimated, reducing the difference between observed and predicted ∆^13^C values.

**TABLE 2:** Apparent post‐photosynthetic fractionations between bulk leaf and α‐cellulose in TR δ^13^C (*f*_post_, ‰) and associated standard deviation (SD, ‰) estimated at the eddy‐covariance sites from the AmeriFlux and CarboEuropeFlux networks

Network	Site	Species	Years	*f*_post_	SD
AmeriFlux	US‐Ha1	QURU	2003, 2013	1.97	0.26
		TSCA		6.08	0.25
	US‐Bar	FAGR	2003, 2013	4.32	0.02
		TSCA		5.71	0.68
	US‐DK2	CATO	2002	3.20	
		LITU		3.90	
	US‐Ho1	PIRU	2003, 2013	5.49	0.15
		TSCA		4.08	1.88
	US‐SP1	PIEL	2013	4.85	
		PIPA	2002, 2013	3.64	0.47
	US‐MMS	ACSA	2005	4.36	
		LITU		2.74	
	US‐Slt	PIEC	2013	4.00	
		QUPR		3.00	
	Us‐Fuf	PIPO	2014	3.47	
	Average			4.05	1.14
CarboEuropeFlux	BE‐Bra	PISY_QURO	2001, 2002	1.88	0.14
	IT‐Col	FAGR	2001, 2002	3.00	0.72
	DE‐Hai	FAGR	2001, 2002	1.85	0.38
	FI‐Hyy	ABAL	2001	2.11	
	IT‐Lav	ABAL	2002	3.44	
	FR‐LBr	PIPI	2001, 2002	0.72	0.53
	NL‐Loo	PISY	2001, 2002	2.81	0.42
	DK‐Sor	FAGR	2002	0.65	
	DE‐Tha	PIAB	2001, 2002	3.43	0.28
	Average			2.20	1.04

We thus tested JULES at the AmeriFlux stations using the TR isotopic records corrected with the individual average *f*_post_ values reported in Table 2 for each species and site. Consistent with the results of the global network, predictions using the Prentice model better captured the site‐to‐site variations observed in the isotopic record, especially the lowest ∆^13^C values at the driest site, while also producing average ∆^13^C values generally closer to the observations (Figures S2 and S3). As the rate of carbon assimilated during photosynthesis depends on leaf *c*_i_ (Equation 1 and Equations S1–S3) and photosynthesis and transpiration rates are strongly coupled via leaf stomata (Equations 4 and 14), we, therefore, expected stronger predictive skills of the Prentice stomatal scheme to simulate carbon and water fluxes as measured at the top of the canopy. Consistently, we found that the mean values and interannual variations of GPP and ET measured during the growing season at the AmeriFlux and CarboEuropeFlux stations were better reproduced by JULES using the Prentice model (Pearson's *r* = .5, RMSE = 1.1; Figure S4). Therefore, the Prentice model shows the lowest biases for GPP and ET as well as for the isotopic measurements.

### 3.2 Global variations in Δ^13^C over 1979–2016 and contributions to these changes

**FIGURE 3:**
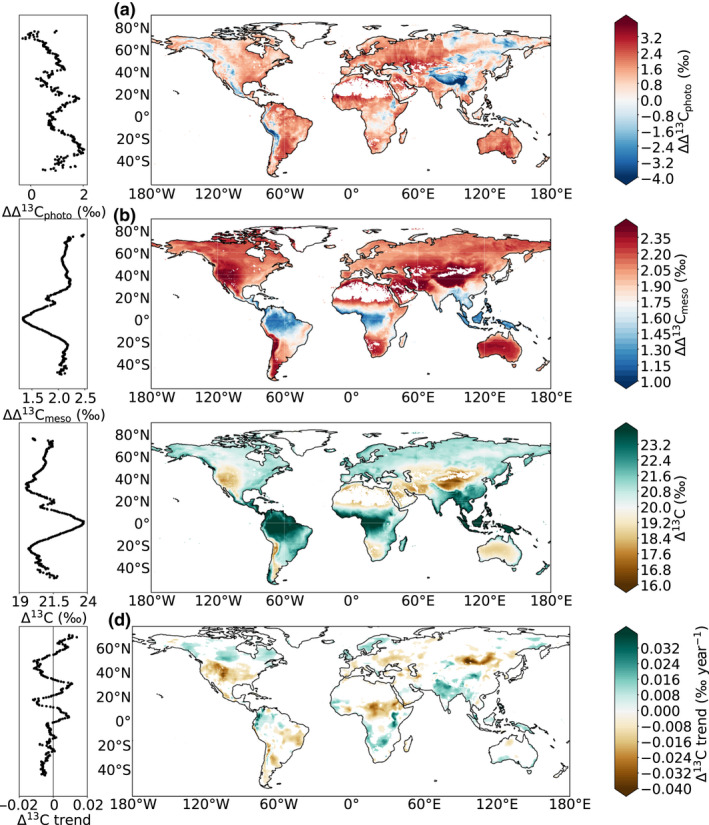
Differences of average Δ^13^C values (a) without and with photorespiratory effect (ΔΔ^13^C_photo_ = Δ^13^C_simple_−Δ^13^C_photo_) and (b) without and with mesophyll effect (ΔΔ^13^C_meso_ = Δ^13^C_photo_−Δ^13^C_photo+meso_) over 1979–2016. Average values (c) and trends (d) in Δ^13^C including both photorespiratory and mesophyll effects over 1979–2016. On the left sides of each panel are the corresponding latitudinal averaged values. In panel (d) only Δ^13^C trends significant at 90% are shown (*p* < .10)

We ran JULES globally using the Prentice stomatal model with the different discrimination models. We found a global average GPP‐weighted Δ^13^C value for forest ecosystems of 23.8 ± 1.9‰ (mean ± SD) using the simple form of the model, 22.9 ± 1.2‰ incorporating the photorespiratory effect, and 20.9 ± 1.5‰ when additionally incorporating the mesophyll effect. There was, however, large spatial variability in the difference of Δ^13^C values with or without the photorespiratory or mesophyll effects. The photorespiratory effect increased average Δ^13^C values in mountainous areas but reduced them in other regions (Figure 3a). The mesophyll effect decreased average Δ^13^C values everywhere across the globe but the effect was smaller in the tropical forest regions and larger in dry and mountainous areas (Figure 3b). The spatial pattern of mesophyll effect resembled that of ET and *T*_r_ (Figure S5), with lower impact on Δ^13^C when (evapo)transpiration was high than when it was low.

The final GPP‐weighted Δ^13^C values (including both photorespiratory and mesophyll effects) varied strongly across the globe, with higher Δ^13^C in regions in tropical forests and lower Δ^13^C in arid and mountainous areas (Figure 3c), consistent with the spatial patterns observed in the leaf isotopic data set (Figure 1c). The trends in Δ^13^C over the 1979–2016 period also differed across regions. Δ^13^C increased in the cold high latitudes of the Northern Hemisphere and/or relatively wet areas such as India but decreased in hot and/or dry regions such as central west United States, Patagonia and eastern Russia (Figure 3d), relatively consistent with the trends inferred from the TR isotopic data set (Figure 2a).

**FIGURE 4:**
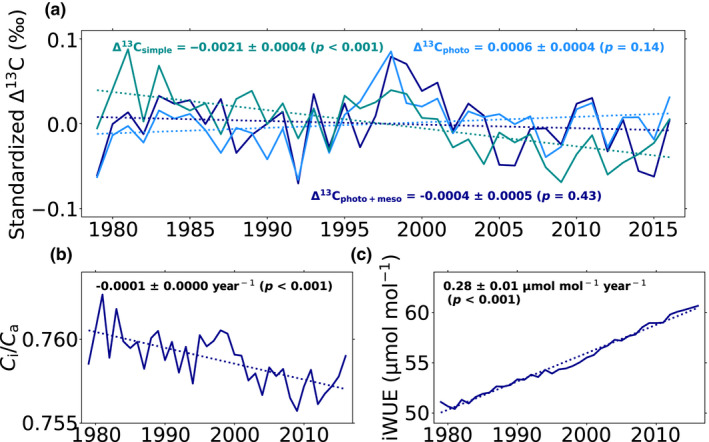
Global average change in (a) Δ^13^C for simple version of the discrimination model (dark green), with photorespiratory effect (light blue) and with both photorespiratory and mesophyll effects (dark blue), (b) *c*_i_/*c*_a_ and, (c) iWUE over 1979–2016. The trends and associated standard deviations and P‐value of the trends are reported

Globally, annually Δ^13^C decreased slightly over the 1979–2016 period when considering the simple discrimination model (−0.0021 ± 0.0004‰ year^−1^, *p* < .001; Figure 4a) because of the slight decrease in *c*_i_/*c*_a_ in the global JULES predictions (−0.0001 ± 0.0000 year^−1^, *p* < .001; Figure 4b). However, it stayed relatively constant globally when incorporating the photorespiratory effect and additional fractionation due to the diffusion of CO_2_ within the mesophyll (Figure 4a). The global intrinsic water‐use efficiency (iWUE), which is directly related to *c*_i_/*c*_a_ and thus Δ^13^C, increased by 0.28 ± 0.01 µmol mol^−1^ year^−1^ over the same period (Figure 4c).

**FIGURE 5:**
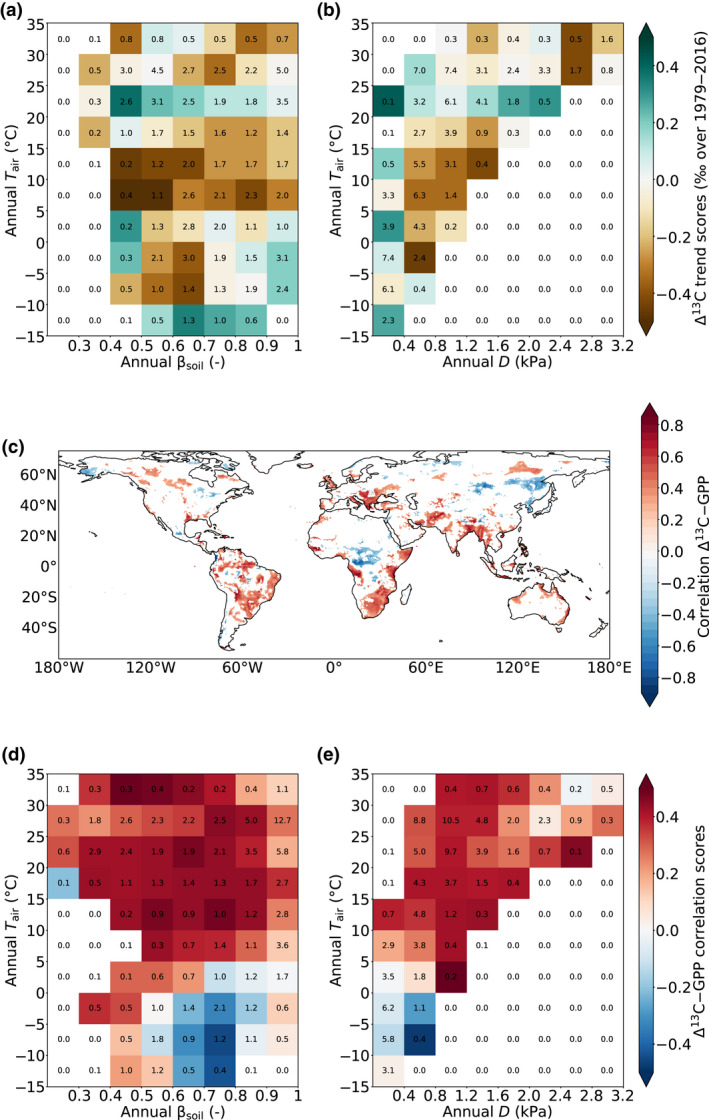
(a, b) Δ^13^C trend scores (‰ over the whole 1979–2016 period) and (d, e) Δ^13^C‐GPP correlation scores for groups of sites with different ranges of annually average *T*_air_ and *β*_soil_ values (a, d) or *D* values (b, e) over 1979–2016. (c) Correlation map between Δ^13^C and GPP showing only correlations significant at 90% (*p* < .10). The scores are calculated as the average of the Δ^13^C trends (a, b) or Δ^13^C‐GPP correlations (d, e) within each group. The black numbers in the middle of each square correspond to the percentage of data within the group. Only groups with more than 20 grid‐points are considered

When combining the Δ^13^C trends within different groups of climatic regions (Figure 5a,b), we did not find clear pattern of variation over the globe, except in temperate regions (annual *T*_air_ of 5–20°C) where Δ^13^C tended to decrease (up to −0.4‰ over the whole 1979–2016 period), and in cold and wet regions where it increased. In contrast, GPP stayed constant or increased almost everywhere in the globe (Figure S6a), but the rate of increase was slightly lower in cold regions with annual *T*_air_ lower than 5°C (Figure S6b,c).

### 3.3 Sign, magnitude, and drivers of Δ^13^C‐GPP relationships over 1979–2016

Large variations in the sign and magnitude of the Δ^13^C‐GPP relationship were observed between regions (Figure 5c), with negative correlations in tropical Africa or in high northern latitudes such as in Alaska, northern Eurasia and eastern Russia, but positive correlations elsewhere. When combining the Δ^13^C‐GPP grid‐point correlations within the different groups of climatic regions, we found that Δ^13^C and GPP were positively related in most environments except in wet‐humid and cold areas (annually averaged *T*_air_ < 5°C and *β*_soil_ > 0.6 or *D* < 0. 8 kPa) and in dry‐warm region (annually averaged *T*_air_ = 10–15°C and *β*_soil_ < 0.3) where they tended to be negatively correlated (Figure 5d,e).

The multiple linear regression models showed that Δ^13^C and GPP values tended to increase with *T*_air_ but decrease with *D* in most groups of Δ^13^C‐GPP correlation, with atmospheric CO_2_ only slightly contributing to modulate Δ^13^C and GPP (Table 3). However, while Δ^13^C was predominantly influenced by *D* variations, GPP was mainly driven by *T*_air_. Around 0.8 ± 0.9% and 55.4 ± 6.3% of the variance in GPP was explained by *D* and *T*_air_, respectively, in group of sites where Δ^13^C and GPP were negatively related, but only 9.5 ± 0.4% and 25.6 ± 19.5% of GPP variability was explained by the two drivers, respectively, in the other groups. These results suggest that *T*_air_ had a stronger influence on GPP in cold‐wet and dry‐warm areas. In contrast, 35.2 ± 6.0% and 5.5 ± 0.5% of the variance in Δ^13^C was explained by *D* and *T*_air_, respectively, in sites with negative Δ^13^C‐GPP correlations, while 31.8 ± 8.2% and 26.2 ± 4.5% was explained by the two drivers, respectively, in sites with positive correlations.

**TABLE 3:** Summary statistics for the environmental dependencies of Δ^13^C (‰) and gross primary production (GPP) (gC m^−2^ s^−1^) within groups of Δ^13^C‐GPP correlations. Standardized fitted coefficients of the linear regression models are reported with percentage of the variance explained by each fixed effect in parenthesis. The coefficient of determination (*R* ^2^) is also shown

Correlation group	Climatic range	Variable	CO_2_	*T*_air_	*D*	*R* ^2^
Negative	*T*_air_ < 5°C and *β*_soil_ > 0.6	Δ^13^C	0.01 (<0.01%)	0.25 (5.8%)	−0.63 (35.2%)	.41
		GPP	0.16 (2.7%)	0.89 (62.1%)	0.06 (0.3%)	.65
	*T*_air_ = 15–20°C and *β*_soil_ < 0.3	Δ^13^C	0.01 (<0.01%)	<0.01 (4.8%)	−0.62 (29.2%)	.34
		GPP	0.04 (2.3%)	0.66 (54.4%)	−0.15 (0.3%)	**.57**
Positive	*T*_air_ ≥ 5°C or *β*_soil_ ≤ 0.6	Δ^13^C	0.01 (<0.01%)	1.42 (23.0%)	−1.51 (26.0%)	.49
		GPP	0.23 (0.4%)	3.05 (39.4%)	−1.47 (9.2%)	.49
Negative	*T*_air_ < 5°C and *D* ≤ 0.8 kPa	Δ^13^C	0.01 (<0.01%)	0.28 (5.8%)	−0.75 (41.2%)	.47
		GPP	0.16 (1.38%)	1.17 (49.7%)	−0.23 (1.9%)	.53
Positive	*T*_air_ ≥ 5°C or *D* > 0.8 kPa	Δ^13^C	0.01 (<0.01%)	1.49 (29.4%)	−1.69 (37.6%)	.67
		GPP	0.26 (0.5%)	1.55 (11.8%)	−1.41 (9.7%)	.22

## 4 DISCUSSION

Examining decadal variations in Δ^13^C of C_3_ woody plants across the globe and their link to GPP is important for understanding how forest ecosystems have adjusted their physiology with environmental changes over the recent historical period. Previous studies have suggested that (1) Δ^13^C increased globally at least since 1978 (Keeling et al., 2017) and (2) Δ^13^C and GPP are positively related to each other (Belmecheri et al., 2014; Peters et al., 2018). Our results based on a new carbon isotopic capability in the land‐surface model JULES challenge these assumptions showing that not only Δ^13^C trends varies across regions and stayed nearly constant globally over the past decades but that the directionality of the relationship between Δ^13^C and GPP strongly depends on the climatic regions considered.

### 4.1 Δ^13^C values and trends vary across regions as a response to environmental conditions

The average Δ^13^C values predicted by JULES vary depending on the discrimination model considered, with almost 3‰ of difference in Δ^13^C between the simple and the full models. The large variability in the photorespiratory effect on Δ^13^C values observed across the globe mainly reflects the impact of atmospheric pressure (and thus elevation) on the photorespiratory term (i.e., −*f*Γ*/*c*_a_, Equation 5), while the one of the mesophyll effect is related to the impact of evaporative demand on the mesophyll term (i.e., −*a*_m_(*c*_i_ − *c*_c_)/*c*_a_, Equation 5). When exposed to drier conditions, plants reduce both their stomatal (*g*_sc_) and mesophyll (*g*_m_) conductance (Dewar et al., 2018; Knauer et al., 2020) to minimize water loss during transpiration but at the expense of carbon gain. The reduction of *g*_m_ in addition to *g*_sc_ with water stress may reduce Δ^13^C values in drier environments, resulting in a larger mesophyll effect on Δ^13^C in dry areas. In contrast, in tropical forests around 20°N and 20°S where the (evapo)transpiration is important, the mesophyll effect is smaller.

Overall, trends in Δ^13^C varied widely across the globe, with tendencies towards decreasing in temperate and warm regions but increasing in some cold and/or wet areas. Varying temporal patterns of Δ^13^C trends were also reported in the literature (Andreu‐Hayles et al., 2011; Keller et al., 2017; Lavergne et al., 2017; Martinez‐Sancho et al., 2018), but there were never clearly associated to site environmental conditions. Contrasting Δ^13^C trends between tree species growing in the same sites have also been observed (Guerrieri et al., 2016; Levesque et al., 2017), which may reflect differences in ecophysiological characteristics between species. These potential differences in Δ^13^C trends between PFTs were not predicted by JULES because no fixed parameter defined the behavior of forest PFTs in the Prentice stomatal model. Our results show that the sensitivity of Δ^13^C to rising CO_2_ is modulated by climate variations and that no single scenario of physiological response to CO_2_ (e.g., following theoretical framework proposed by Saurer et al. (2004)) can explain this pattern.

### 4.2 Global predicted trend in Δ^13^C and its contributions differ from Keeling et al. (2017) study

Our simulations using the Prentice model and including discrimination effects from photorespiration and mesophyll conductance suggest that Δ^13^C stayed relatively constant globally during the 1979–2016 period. Our estimate contrasts with Keeling et al. (2017) findings (referred to as K2017 hereafter), which reported a global increase of Δ^13^C of 0.014 ± 0.007‰ ppm^−1^ over 1978–2014 based on atmospheric measurements and a box model. In K2017, the authors assumed a constant Γ*, though it has been shown that Γ* depends on atmospheric pressure and leaf temperature (Bernacchi et al., 2002, 2003). For instance, the increase in global *T*_air_ of 0.029 ± 0.007°C year^−1^ over 1978–2014 may have led to an increase of Γ* of 0.055 ± 0.014 ppm year^−1^ for a range of leaf temperature values of 17–25°C. Such an increase in Γ* counteracted the positive effect of rising CO_2_ (1.72 ± 0.05 ppm year^−1^) on Δ^13^C leading to a lower contribution from the photorespiratory effect to the global Δ^13^C trend estimated by JULES than by K2017 study (i.e., 0.0015 ± 0.0050‰ ppm^−1^ vs. 0.0041 ± 0.0014‰ ppm^−1^).

The authors in K2017 also based their calculations on a linear increase of *A* of 45% for a doubling of CO_2_ as suggested by Franks et al. (2013) (equivalent to 6.3% increase for the range of CO_2_ values from 330 to 377 ppm over 1978–2014), but they assumed constant global mean *g*_m_ value over time, while *g*_m_ is expected to decrease with rising atmospheric CO_2_ (Flexas et al., 2012; Knauer et al., 2020). As a result, the authors found a contribution from the mesophyll effect to the global increasing Δ^13^C trends of 0.006 ± 0.003‰ per ppm increase of CO_2_ (using a mesophyll term defined as −(*b* − *a*_m_)/*g*_m_ *A*/*c*_a_, with *A* = 9 µmol m^−2^ s^−1^ and *g*_m_ = 0.2 mol m^−2^ s^−1^). This estimate strongly differs from that predicted by JULES where the mesophyll effect reduces the global trend in Δ^13^C by −0.0006 ± 0.001‰ ppm^−1^. Using a lower *g*_m_ value at 377 ppm of CO_2_ than that used by K2017 gives a mesophyll contribution of around 0.002‰ ppm^−1^ with *g*_m_ = 0.19 mol m^−2^ s^−1^ but of −0.003‰ ppm^−1^ with *g*_m_ = 0.18 mol m^−2^ s^−1^, more in line with our JULES estimate. Thus, the assumption made in K2017 about the *g*_m_ value strongly influenced their mesophyll contribution estimate and may have biased their interpretations.

Our modelled and TR‐based estimates of Δ^13^C trends suggest that changes in Δ^13^C of C_3_ plants cannot account for an increase in apparent biospheric discrimination as suggested by K2017. If there were changes to biosphere‐atmosphere isotopic fluxes over the last several decades with an effect on the global δ^13^CO_2_ trend that resembled an apparent increase in Δ^13^C, they must rather derive from non‐photosynthetic processes, such as changes in post‐photosynthetic fractionation, changes in species abundance and distribution or in the proportion of C_3_:C_4_ plant productivity, or changes in the residence time of carbon in plants or soils.

### 4.3 The negative Δ^13^C‐GPP correlation in cold‐wet and tropical African forests reflects the strong influence of *T*_air_ on photosynthesis in these environments

Overall, Δ^13^C and GPP tended to be negatively correlated in wet‐cold regions and tropical African forests (where Δ^13^C remained constant or decreased, while GPP increased over 1979–2016; see also Figures 3d and S6a), but were positively related elsewhere. Because Δ^13^C and A vary in similar directions with changes in *c*_i_/*c*_a_ and Γ*/*c*_a_ (see also Text S2), increasing with rising *c*_a_ and *T*_air_ but decreasing with rising *D*, we would expect Δ^13^C and canopy‐scale A (i.e., GPP) to be positively correlated everywhere. Given that *T*_air_ and *D* are positively related to each other (Pearson's *r* = .70, *p* < .001), it is possible that the dominant influences of *T*_air_ on GPP (via A) and of *D* on Δ^13^C in particular in cold and wet‐humid regions both contributed to the decoupling between Δ^13^C and GPP. The stronger impact of *T*_air_ on GPP than on Δ^13^C could be due to the additional effect of rising *T*_air_ on the maximum carboxylation capacity (*V*_cmax_) or electron transport (*J*_max_), which do not impact Δ^13^C. It is also possible that it reflects the positive influence of increasing *T*_air_ on the leaf area (Luyssaert et al., 2007; Piao et al., 2006) or on the growing season length which promotes carbon uptake in those regions (Cai & Prentice, 2020; Piao et al., 2009).

The spatial distribution of the Δ^13^C‐GPP correlations found in European forests (i.e., negative north of 60°N but positive elsewhere) is consistent with Shestakova et al. (2019) results in the region using Δ^13^C and TR growth. The authors of this study also found a change from negative to positive correlations in the Scandinavian area over the course of the 20th century very likely due to increasing drying. Their results suggest that if the atmospheric drying observed over the past century (Grossiord et al., 2020) was to continue, it is possible that the Δ^13^C‐GPP relationship in relatively wet and cold regions may switch from negative to positive because of the increasing influence of *D* on GPP. Thus, studying variations in Δ^13^C and GPP together may help documenting the areas where the drivers of photosynthesis could change in the coming decades due to climate change.

### 4.4 Limitations of the study

We acknowledge several limitations in our approach to examining Δ^13^C mean values and trends and Δ^13^C‐GPP relationships. First, the Δ^13^C values across the globe predicted by JULES (including both photorespiratory and mesophyll effects) were around 0.8‰ higher in average than those derived from the global leaf network, and up to 1.6‰ higher than those from the TR network. In addition, the magnitudes of the Δ^13^C trends were almost four time lower in the predictions than in the TR isotopic network and the IAV in Δ^13^C was largely underestimated by the model. The apparent model bias in Δ^13^C values may result from uncertainties in both the predicted and observation‐derived Δ^13^C.

On the one hand, it is possible that the ‘least‐cost’ Prentice model overestimate *c*_i_ (or *c*_c_) because it ignores the effect of soil drying on stomata aperture, leading to an overestimation of Δ^13^C. A recent study showed that the ratio of *c*_i_ to *c*_a_ could be reduced by up to 2% globally when considering the impact of soil water stress on the stomatal activities (Lavergne, Sandoval, et al., 2020). These findings suggest that *c*_i_ (and *c*_c_) should decrease with a reduction of plant‐available soil water, resulting in lower predicted Δ^13^C values in particular in dry areas, more in line with the observed values. The use of yearly average (instead of monthly) *c*_a_ values to predict Δ^13^C may also have led to an overestimation of the simulated Δ^13^C values because of the strong seasonal cycle of *c*_a_ (i.e., lower *c*_a_ values in summer than in winter). Given that the seasonal variations in *c*_a_ have increased since the 1950s in particular in the northern high latitudes (Graven et al., 2013) but were not accounted for here, we could expect the IAV in Δ^13^C to be lower in the predictions than in the TR observations. Finally, we assumed a constant *g*_m_/*g*_s_ following recent studies (Flexas & Carriqui, 2020; Yiotis & McElwain, 2019) to estimate *c*_c_ (Equation (1), (9a), (9b)) while it is still unclear whether *g*_m_ responds differently or more strongly than *g*_s_ to environmental changes. For instance, (all else equal) if *g*_m_/*g*_s_ increases (e.g., *g*_m_ changes at a higher rate than *g*_s_), *c*_c_ (and thus *c*_c_/*c*_a_) should decrease, resulting in an even more negative effect of *g*_m_ on Δ^13^C trend. In contrast if *g*_m_/*g*_s_ decreases (e.g., *g*_s_ changes at a higher rate than *g*_m_), *c*_c_ (and thus *c*_c_/*c*_a_) should increase, reducing the negative effect of *g*_m_ on Δ^13^C trend. Thus, our assumption of a constant *g*_m_/*g*_s_ may have affected the predicted Δ^13^C values. Nevertheless, a recent study found no significant difference in *g*_m_/*g*_s_ values across long‐term drought‐stress treatments (Ma et al., 2021), increasing confidence about our assumption.

On the other hand, biases in the observation‐derived Δ^13^C may have increased the discrepancies between predicted and observed Δ^13^C values. Since the yearly average δ^13^CO_2_ values used to calculate Δ^13^C from the leaf and TR isotopic observations are lower than those during the growing season, the data‐derived Δ^13^C values may have been overestimated at least in the Northern Hemisphere where the δ^13^CO_2_ seasonal cycle varies between 0.2‰ in tropical areas to up to 0.8‰ in high latitudes (Scripps CO_2_ program). Also, we assumed a constant *f*_post_ to correct the TR isotopic timeseries, while our analyses based on isotopic data from the AmeriFlux stations suggest that *f*_post_ varies strongly across sites, species and even years, and may be in average twice higher than the standard *f*_post_ values often used in the literature (4.1‰ vs. 2.1‰). Using the higher *f*_post_ estimates would result in higher TR‐derived ∆^13^C values, reducing the difference between observed and predicted ∆^13^C values, and thus the apparent bias in the predictions.

Despite all the above‐mentioned limitations, the spatial patterns of Δ^13^C mean values predicted by JULES were in good agreement with the leaf isotopic observations and with Cornwell et al. (2018) study based on a data‐driven statistical approach, suggesting that the model captures relatively well the spatial variability in Δ^13^C values across the globe. Since the average Δ^13^C values inferred from the JULES simulations for the TR sites decreased over 1979–2016 (−0.006‰ year^−1^, *p* < .001), whereas those estimated from the observed TR Δ^13^C timeseries stayed nearly constant (*p* = .097), it is, however, possible that JULES has a negative bias in the global trend in Δ^13^C. One reason for this bias may be that the slight decrease in the predicted *c*_i_/*c*_a_ values over 1979–2016 (reduction of 0.5%, *p* < .001) is not realistic and that *c*_i_/*c*_a_ should rather stay constant over time as suggested by other studies (Frank et al., 2015; Wong et al., 1979). Nevertheless, JULES predicted an almost proportional increase of iWUE compared to atmospheric CO_2_ over 1979–2016 (21% vs. 20%, respectively), consistent with the rate of increase estimated by recent studies for similar periods (Adams et al., 2020; Lavergne et al., 2019; Mathias & Thomas, 2021; Soh et al., 2019), suggesting that despite biases to predict IAV and trend in Δ^13^C, the carbon uptake per unit of water lost is relatively well constrained in JULES.

## 5 CONCLUSIONS

This is the first study investigating decadal changes in Δ^13^C and documenting the links between Δ^13^C and GPP across the globe using a data‐model approach. Δ^13^C predicted by JULES increases in some cold and wet environments but decreases in temperate regions over the 1979–2016 period. These patterns of variations show the strong coupling between the water and carbon cycles across the globe. Globally, predicted Δ^13^C stayed nearly constant over the studied period, which differs from the large secular increase in Δ^13^C reported in K2017 based on atmospheric measurements. The difference between K2017 and this study mainly lies on the contrasting simulated contributions from photorespiratory and mesophyll effects to the global trend. Our results suggest that studying Δ^13^C and GPP together may inform about the most influential drivers of photosynthesis and, thus, help understanding temporal changes in the land carbon uptake.

## AUTHOR CONTRIBUTIONS

A.L. designed the research, analyzed the data, and wrote the first draft. D.H. and R.G. provided the stable carbon isotope data from leaves and TRs in the CarboEuropeFlux and AmeriFlux stations, respectively. R.J.O. helped in the setup and run of JULES on the NERC JASMIN platform using the Rose/Cylc suite control system. I.C.P. and H.G. provided additional suggestions on the research plans. All co‐authors contributed to improve the manuscript.

## CONFLICT OF INTEREST

All authors declare that they have no conflicts of interest.

## CODE AVAILABILITY STATEMENT

The model code and the files needed for running it are available from the Met Office Science Repository Service (MOSRS; https://code.metoffice.gov.uk/trac/jules/; registration required). The results presented in this paper were obtained by running JULES vn5.6 branch with a new carbon isotopic modelling capability (code available after registration with MOSRS at https://code.metoffice.gov.uk/trac/jules/browser/main/branches/dev/alienorlavergne/vn5.6_jules_Cisotopes). The runs were performed with the Rose suite u‐bx886 (https://code.metoffice.gov.uk/trac/roses‐u/browser/b/x/8/8/6). The Python code developed to create the main manuscript figures is available at https://github.com/Alielav/GCB_Lavergneetal_2021.

## Supporting information

Supplementary Material

Supplementary Material

## Data Availability

The isotopic data that support the findings of this study are available in the supplementary material of this article. The FLUXNET2015 data used to test JULES are available for download at https://fluxnet.org/data/fluxnet2015‐dataset/. The model outputs for the individual sites are available at https://github.com/Alielav/GCB_Lavergneetal_2021, while those for the global run are available upon request through the NERC JASMIN platform (http://www.jasmin.ac.uk/).
