# Supplementary material for: Global decadal variability of plant carbon isotope discrimination and its link to gross primary production
Source: Glob Chang Biol. 2021 Oct 18;28(2):524–41. doi: 10.1111/gcb.15924 (PMC9298043; doi:10.1111/gcb.15924)
Supplement: Supplementary file 1 — Supplementary Material [file GCB-28-524-s002.pdf]

Global Change Biology - Supporting Information

Article: Global decadal variability of plant carbon isotope discrimination and its link to gross primary production

Authors: Aliénor Lavergne, Deborah Hemming, Iain Colin Prentice, Rossella Guerrieri, Rebecca J. Oliver, and Heather Graven

Texts S1-S2, Figures S1-S6, Tables S1-S3

Text S1 Limiting factors of photosynthesis in Farquhar et al. (1980) model

The potential rate of carbon assimilation during photosynthesis (A_p) in the Farquhar et al. (1980) model is the minimum of two limiting factors (electron transport A_J and carboxylation rate A_C).

Under light limitation, A_J is defined as:

$$A_J = \frac{J}{4}\frac{(\chi-\gamma)}{(\chi+2\gamma)} \quad \text{(S1)}$$

χ is the ratio of leaf internal (c_i) to ambient (c_a) CO_2 (µmol mol^{-1}), and γ is the ratio of photorespiratory CO_2 compensation point (Γ^*, µmol mol^{-1}) to c_a (µmol mol^{-1}). J is the rate of electron transport (µmol m^{-2} s^{-1}) defined as:

$$J = \frac{\left(\alpha Q + J_{max} - \sqrt{(\alpha Q + J_{max})^2 - 4\,\theta\,\alpha Q\,J_{max}}\right)}{2\,\theta} \quad \text{(S2)}$$

Q is the incident photosynthetically active photon flux density (mol photon m^{-2} s^{-1}), α is the quantum yield of electron transport (equal to 0.34 mol electrons mol^{-1} PAR photon). J_{max} is the maximum electron-transport rate (µmol m^{-2} s^{-1}) and θ the curvature of the light response (equal to 0.90).

Under RuBisCO limitation, A_C is defined as:

$$A_c = V_{cmax}\frac{(\chi-\gamma)}{(\chi+\kappa)} \quad \text{(S3)}$$

κ is the ratio of the effective Michaelis constant (K) to c_a (µmol mol^{-1}), and V_{cmax} is the maximum rate of carboxylation (µmol m^{-2} s^{-1}).

V_{cmax} and J_{max} depend on the leaf temperature (T_k) in K following an Arrhenius function and a peaked function as in Medlyn et al. (2002):

$$f(T_k) = exp^{\left(\frac{Ha(T_k-298)}{298\,R\,T_k}\right)} \frac{1+exp^{\left(\frac{298\,\Delta S-H_d}{298\,R}\right)}}{1+exp^{\left(\frac{T_k\,\Delta S-H_d}{T_k\,R}\right)}} \qquad \text{(S4)}$$

where H_a and H_d are the rates of exponential increase and decrease of the function below and above the optimum, respectively (or activation and deactivation energies, kJ mol^{-1}). ΔS is the entropy factor (J mol^{-1} K^{-1}). The values of H_a, H_d and ΔS for V_{cmax} and J_{max} depend on the plant functional type (PFT, see Table S1). R is the universal constant (8.314, J mol^{-1} K^{-1}).

V_{cmax} and J_{max} are then calculated from their respective values at 25°C (µmol m^{-2} s^{-1}), for each PFT as:

$$V_{cmax} = V_{cmax,25}\; f_{Vcmax}(T_k) \qquad \text{(S5)}$$

$$J_{max} = V_{cmax,25}\; f_{Jmax}(T_k) \frac{J_{max,25}}{V_{cmax,25}} \qquad \text{(S6)}$$

The ratio of $J_{max,25}$ to $V_{cmax,25}$ is set constant and depends on the PFT considered. $V_{cmax,25}$ is calculated as in Medlyn et al. (1999):

$$V_{cmax,25} = i_v + s_v\, N_a \qquad \text{(S7)}$$

N_a is the leaf nitrogen content per unit area (kgN m^{-2}), and i_v (µmol m^{-2} s^{-1}) and s_v (µmol gN^{-1} s^{-1}) are the intercept and slope of the relationship between $V_{cmax,25}$ and N_a, respectively, which depend on the PFT considered (Kattge et al., 2009). N_a is calculated as the product of PFT-dependent leaf traits following Harper et al. (2016):

$$N_a = N_{mass}\, LMA\; LAI \qquad \text{(S8)}$$

N_{mass} is the top-leaf nitrogen mass per leaf mass (kgN kg^{-1}), LMA is the leaf mass per unit area (kg m^{-2}) and LAI is leaf area index (m^2 m^{-2}).

All the PFT-dependent parameters in JULES are reported in Table S1.

Text S2 Similar influence of χ and γ on $\Delta^{13}C$ and on the limiting rates of photosynthesis (A_C and A_J)

Assuming infinite mesophyll conductance (g_m), $\Delta^{13}C$ can be written as:

$$\Delta^{13}C = a + (\bar{b} - a)\,\chi - f\gamma \qquad \text{(S9a)}$$

where a, $\bar{b}$ and f are the isotope fractionations due to CO_2 diffusion across the stomata (4.4‰), effective RuBisCO carboxylation (28‰) and photorespiration (12‰). χ and γ are as defined above: $\gamma = \frac{\Gamma^*}{c_a}$ and $\chi = \frac{c_i}{c_a}$.

Assuming finite g_m, $\Delta^{13}C$ is:

$$\Delta^{13}C = a + (b - a)\,\chi_c - f\gamma + a_m(\chi - \chi_c) \qquad \text{(S9b)}$$

where a_m is the isotope fractionations due to CO_2 diffusion across the mesophyll cell (1.8‰), b is equivalent to $\bar{b}$ assuming finite g_m (30‰) and χ_c is the ratio of chloroplastic (c_c) to ambient (c_a) partial pressure of CO_2 (with $c_c < c_i$).

The derivatives of $\Delta^{13}C$ with γ and χ when assuming infinite g_m (Eqn S9a) are:

$$\frac{\partial \Delta^{13}C}{\partial \gamma} = -f < 0 \qquad \text{(S10a)}$$

$$\frac{\partial \Delta^{13}C}{\partial \chi} = \bar{b} - a > 0 \qquad \text{(S10b)}$$

The derivatives of $\Delta^{13}C$ with γ, χ and χ_c when assuming finite g_m (Eqn S9b) are:

$$\frac{\partial \Delta^{13}C}{\partial \gamma} = -f < 0 \qquad \text{(S11a)}$$

$$\frac{\partial \Delta^{13}C}{\partial \chi} = a_m > 0 \qquad \text{(S11b)}$$

$$\frac{\partial \Delta^{13}C}{\partial \chi_c} = b - a - a_m > 0 \qquad \text{(S11c)}$$

Thus, $\Delta^{13}C$ increases with rising χ (or χ_c) but decreases with rising γ.

The derivatives of log-transformed A_J and A_C (Equations 1 and 2) with γ and χ are:

$$\frac{\partial LnA_J}{\partial \gamma} = -\frac{3\chi}{(\chi-\gamma)(\chi+2\gamma)} < 0 \qquad \text{(S12a)}$$

$$\frac{\partial LnA_J}{\partial \chi} = \frac{3\gamma}{(\chi-\gamma)(\chi+2\gamma)} > 0 \qquad \text{(S12b)}$$

and

$$\frac{\partial LnA_C}{\partial \gamma} = -\frac{(2\chi+K-\gamma)}{(\chi-\gamma)(\chi+\kappa)} < 0 \qquad \text{(S13a)}$$

$$\frac{\partial LnA_C}{\partial \chi} = \frac{(\chi+\kappa)}{(\chi-\gamma)(\chi+\kappa)} > 0 \qquad \text{(S13b)}$$

Thus, A_J and A_C increase with rising χ but decrease with rising γ.

The derivatives of $\Delta^{13}C$ and of log-transformed A_J and A_C are always positive with rising χ and negative with rising γ, so $\Delta^{13}C$ and A are expected to vary in similar directions with changing χ and γ.

Table S1 PFT-dependent parameters values of the stomatal conductance and photosynthesis models tested for the five forest PFTs (BET: broadleaf evergreen trees, BDT: broadleaf deciduous trees, NET: needleleaf evergreen trees, NDT: needleleaf deciduous trees).

	**Parameter**	BET-Tr	BET-Te	BDT	NET	NDT
**Jacobs**	f_0 (unitless)	0.875	0.892	0.875	0.875	0.936
	d_{crit} (kg kg^{-1})	0.090	0.090	0.090	0.060	0.041
**Leuning**	a_1 (unitless)	9	9	9	9	9
**Medlyn**	g_1 (mol m^{-2} s^{-1})	5.31	3.37	4.45	2.35	2.35
**Prentice**	β (unitless)	150	150	150	150	150
	β_c (unitless)	170	170	170	170	170
	g_m/g_s (unitless)	1	1	1	1	1
**Farquhar**	$J_{max,25}/V_{cmax,25}$ (unitless)	2.09	1.89	1.93	1.96	1.99
	H_a J_{max} (kJ mol^{-1})	64	35.9	38.8	36.4	38.8
	H_a V_{cmax} (kJ mol^{-1})	86.9	59.6	49.3	63.1	49.3
	H_d J_{max} (kJ mol^{-1})	200	200	200	200	200
	H_d V_{cmax} (kJ mol^{-1})	200	200	200	200	200
	ΔS J_{max} (J mol^{-1} K^{-1})	635	632	663	643	663
	ΔS V_{cmax} (J mol^{-1} K^{-1})	631	634	658	642	658
	LMA (kg m^{-2})	0.1039	0.1403	0.0823	0.2263	0.1006
	N_{mass} (kgN kg^{-1})	0.0170	0.0144	0.0210	0.0115	0.0186
	i_v (µmol m^{-2} s^{-1})	7.21	3.90	5.73	6.32	6.32
	s_v (µmol gN^{-1} s^{-1})	19.22	28.40	29.81	18.15	23.79
	$V_{cmax,25}$ (µmol m^{-2} s^{-1})	41.16	61.28	57.25	53.55	50.83
	f_d (unitless)	0.010	0.010	0.010	0.015	0.015

Table S2 Summary of JULES model experiments at the leaf- to plant- scale

Experiment	Discrimination model		Stomatal model	
JAC_simple	simple	Eqn 15c	Jacobs	Eqn 5
JAC_photo	photorespiration	Eqn 15b		
MED_simple	simple	Eqn 15c	Medlyn	Eqn 6
MED_photo	photorespiration	Eqn 15b		
LEU_simple	simple	Eqn 15c	Leuning	Eqn 7
LEU_photo	photorespiration	Eqn 15b		
PREN_simple	simple	Eqn 15c	Prentice	Eqn 8
PREN_photo	photorespiration	Eqn 15b		
PREN_photomeso	photorespiration + finite mesophyll	Eqn 15a		Eqn 9

Table S3 Summary of CarboEuropeFlux and AmeriFlux stations used in this study

Network	Station	Site	Data source	Lat (°)	Lon (°)	Elev (masl)	EC period	Species	Year leaf isotope
CarboEurope Flux	BE-Bra	Braschaat, Belgium	FLUXNET-2015	51.3	4.5	16	1996-2014	PISY QURO	2001/2002
CarboEurope Flux	DE-Hai	Hainich, Germany	FLUXNET-2015	51.1	10.5	445	2000-2012	FAGR	2001/2002
CarboEurope Flux	DE-Tha	Tharandt, Dresden, Germany	FLUXNET-2015	50.9	13.5	380	1996-2014	PIAB	2001/2002
CarboEurope Flux	DK-Sor	Store Ebberup, Denmark	FLUXNET-2015	55.0	11.6	42	1996-2014	FAGR	2002
CarboEurope Flux	FI-Hyy	Hyytiälä, Finland	FLUXNET-2015	61.5	24.2	170	1996-2014	ABAL	2001/2002
CarboEurope Flux	FR-LBr	Le Bray, Bordeaux, France	FLUXNET-2015	44.7	-0.8	60	1996-2008	PIPI	2001/2002
CarboEurope Flux	IT-Col	Collelongo, Italy	FLUXNET-2015	41.8	13.6	1150	1996-2014	FAGR	2001/2002
CarboEurope Flux	IT-Lav	Lavarone, Italy	FLUXNET-2015	45.9	11.3	150	2003-2014	ABAL	2002
CarboEurope Flux	NL-Loo	Loobos, The Netherlands	FLUXNET-2015	52.1	5.6	25	1996-2013	PISY	2001/2002
AmeriFlux	US-Bar	Bartlett, NH, US	-	44.06	-71.29	272	2004-2018	FAGR TSCA	2003/2013 2003/2013
AmeriFlux	US-Dk2	Duke, NC, US	-	35.97	-79.10	168	2001-2008	LITU CATO	2002 2002
AmeriFlux	US-Fuf	Flagstaff Unmanaged Forest, AZ, US	-	35.09	-111.76	2180	2006-2010	PIPO	2014
AmeriFlux	US-Ha1	Harvard, MA, US	FLUXNET-2015	42.54	-72.17	340	1991-2012	QURU TSCA	2003/2013 2003/2013
AmeriFlux	US-Ho1	Howland, ME, US	-	45.20	-68.74	60	1996-2014	PIRU TSCA	2003/2013 2003/2013
AmeriFlux	US-MMS	Morgan Monroe, IN, US	FLUXNET-2015	39.32	-86.41	275	1999-2014	ACSA LITU	2005 2005
AmeriFlux	US-Slt	Silas Little, NJ, US	-	39.91	-74.60	30	2005-2014	QUPR PIEC	2013 2013
AmeriFlux	US-SP1	Austin Cary, FL, US	-	29.74	-82.22	44	2007-2013	PIPA PIEL	2002/2013 2013

Figure S1 Geographical site locations. (a) Global network of stable carbon isotopic measurements in leaves (brown) and tree rings (TR, black); (b) Eddy-Covariance (EC) flux stations from the CarboEuropeFlux and AmeriFlux networks. Trends in atmospheric vapour pressure deficit (D) over 1979–2016 were estimated using CRU TS 4.02 dataset.

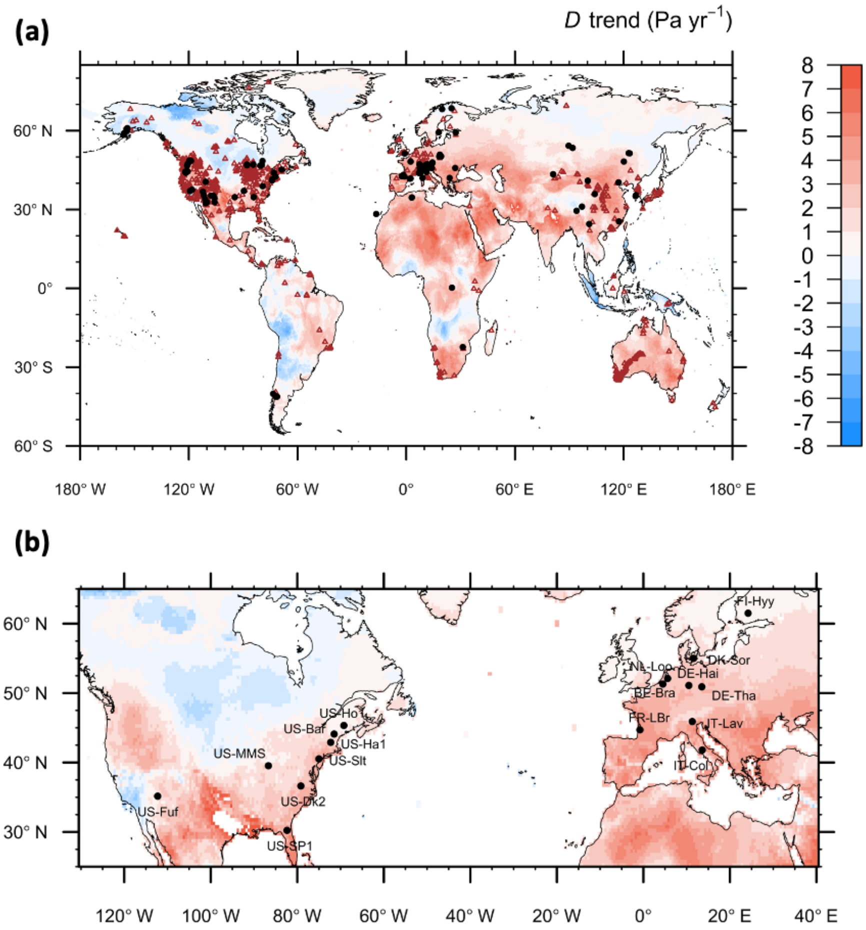

Figure S2 Tree-ring (TR) based $\Delta^{13}C$ timeseries at the eight AmeriFlux stations over the 1982-2012 period compared to the predicted $\Delta^{13}C$ values from the four different stomatal sub-models.

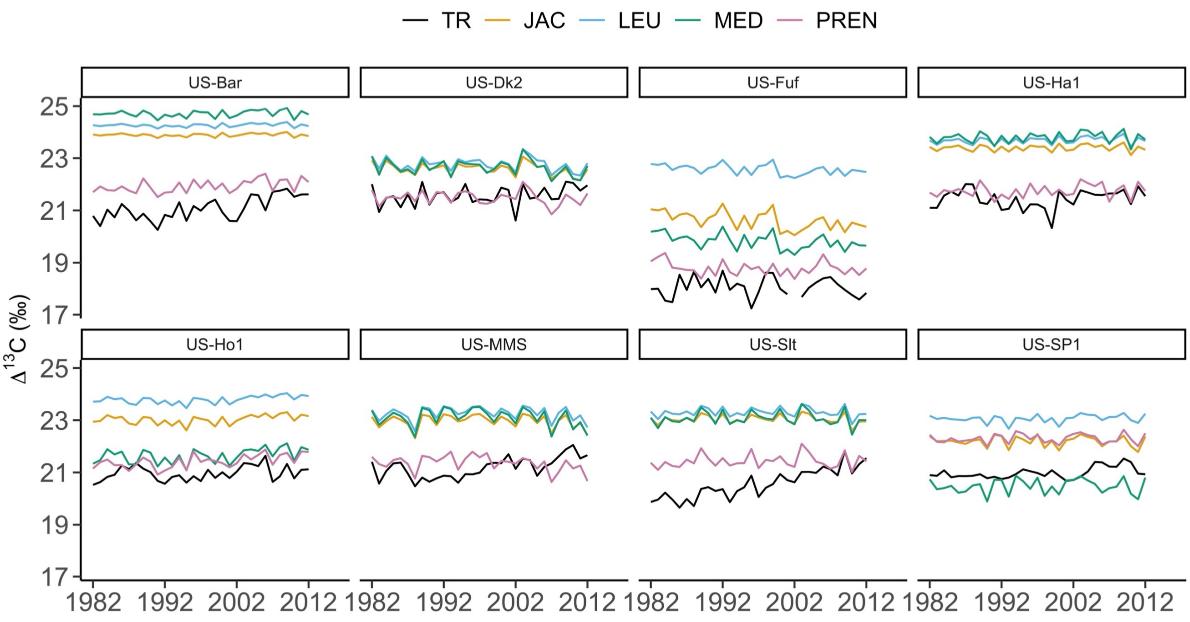

Figure S3 Taylor diagrams of the impacts of stomatal and discrimination representations in JULES on the predicted (a) $\Delta^{13}C$ and (b) iWUE values as compared to the AmeriFlux measurements over the 1982-2012 period.

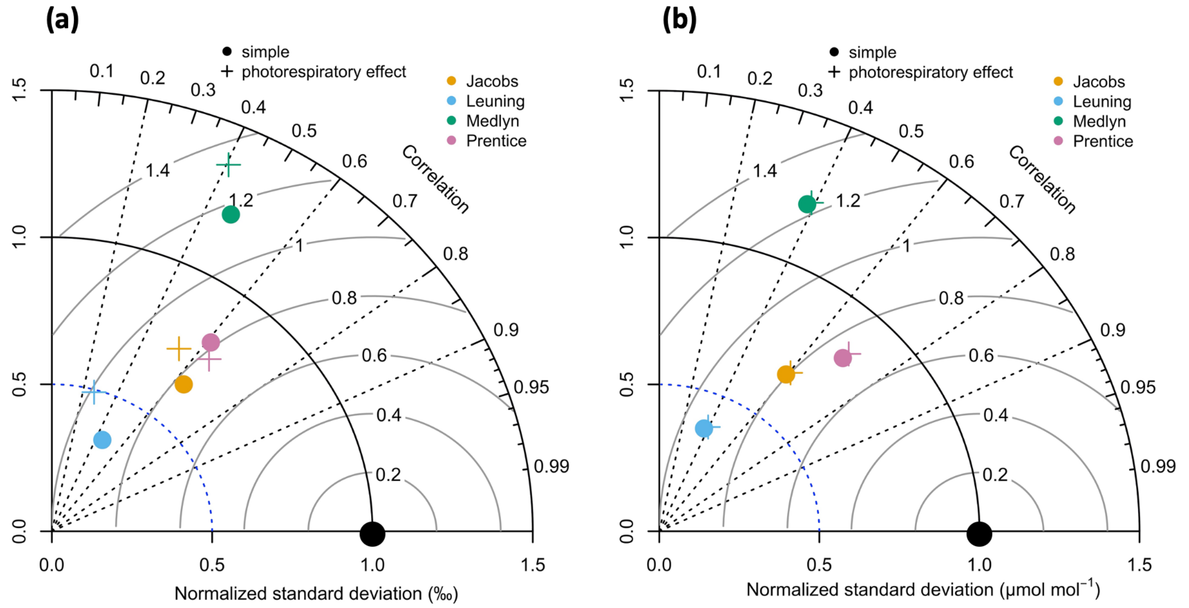

Figure S4 Taylor diagrams of the impacts of the stomatal model chosen on the ecosystem carbon (a, GPP) and water (b, Tr) fluxes at the two AmeriFlux and nine CarboEuropeFlux stations available in the FLUXNET-2015 Tiers 1 network for the growing season (May-September).

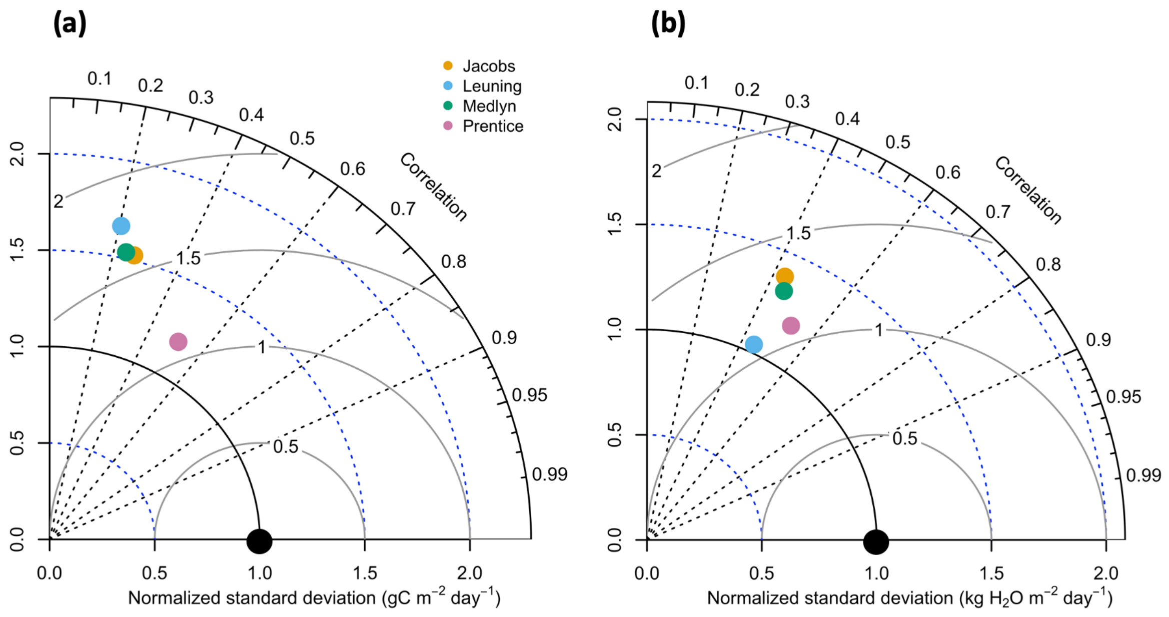

Figure S5 Maps with average values of ET (a) and T_r (b) over 1979-2016. On the left side of the panel are the latitudinal averaged values of the correlations.

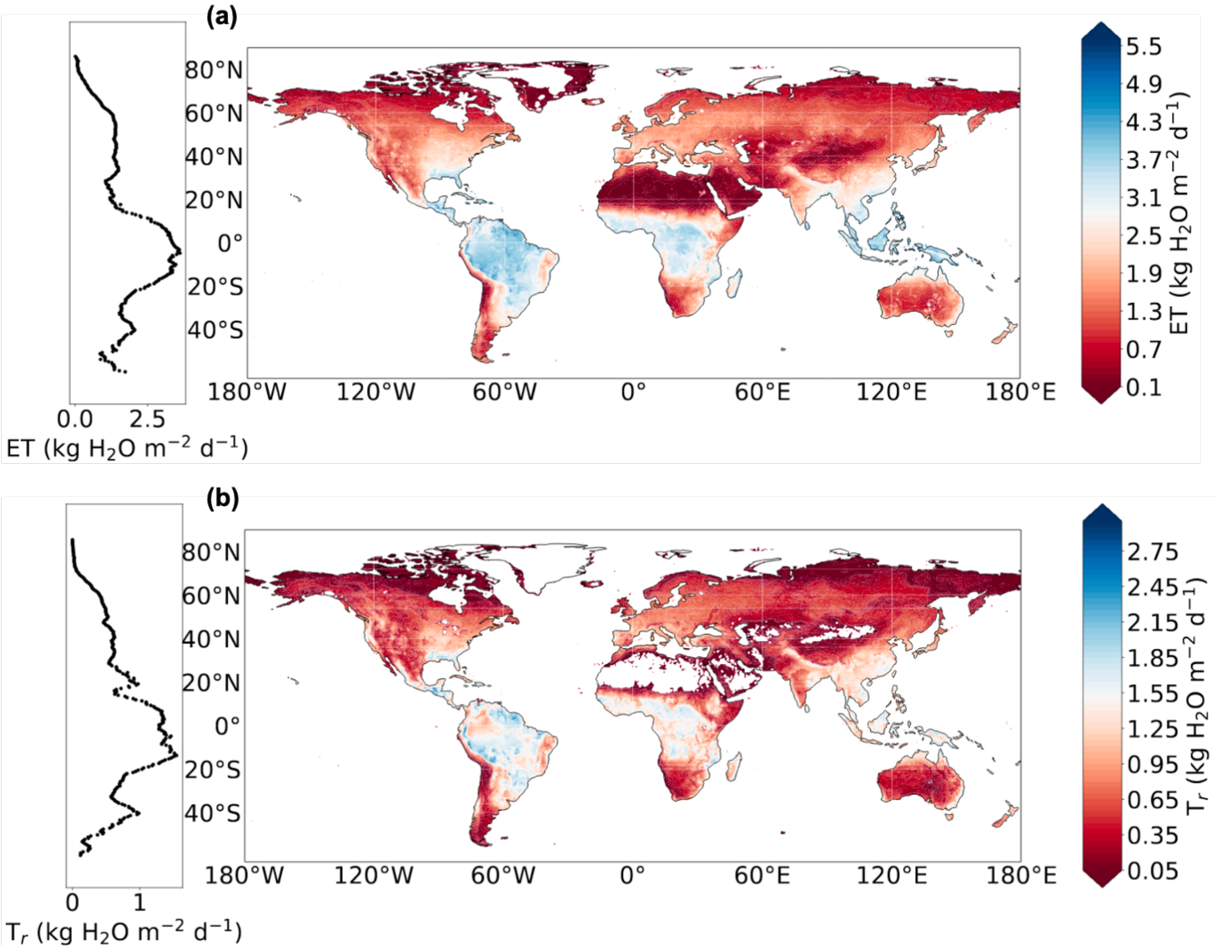

Figure S6 (a) Map of GPP trends (gC m^{-2} d^{-1} yr^{-1}) significant at 90% ($p < 0.10$) over 1979-2016. (b-c) GPP trend scores (gC m^{-2} d^{-1} over the whole 1979-2016 period) for groups of sites with different ranges of annually average T_{air} and β_{soil} values (b) or D values (d) over 1979-2016. The scores are calculated as the average of the GPP trends within each group. The black numbers in the middle of each square correspond to the percentage of data within the group. Only groups with more than 20 grid-points are considered.

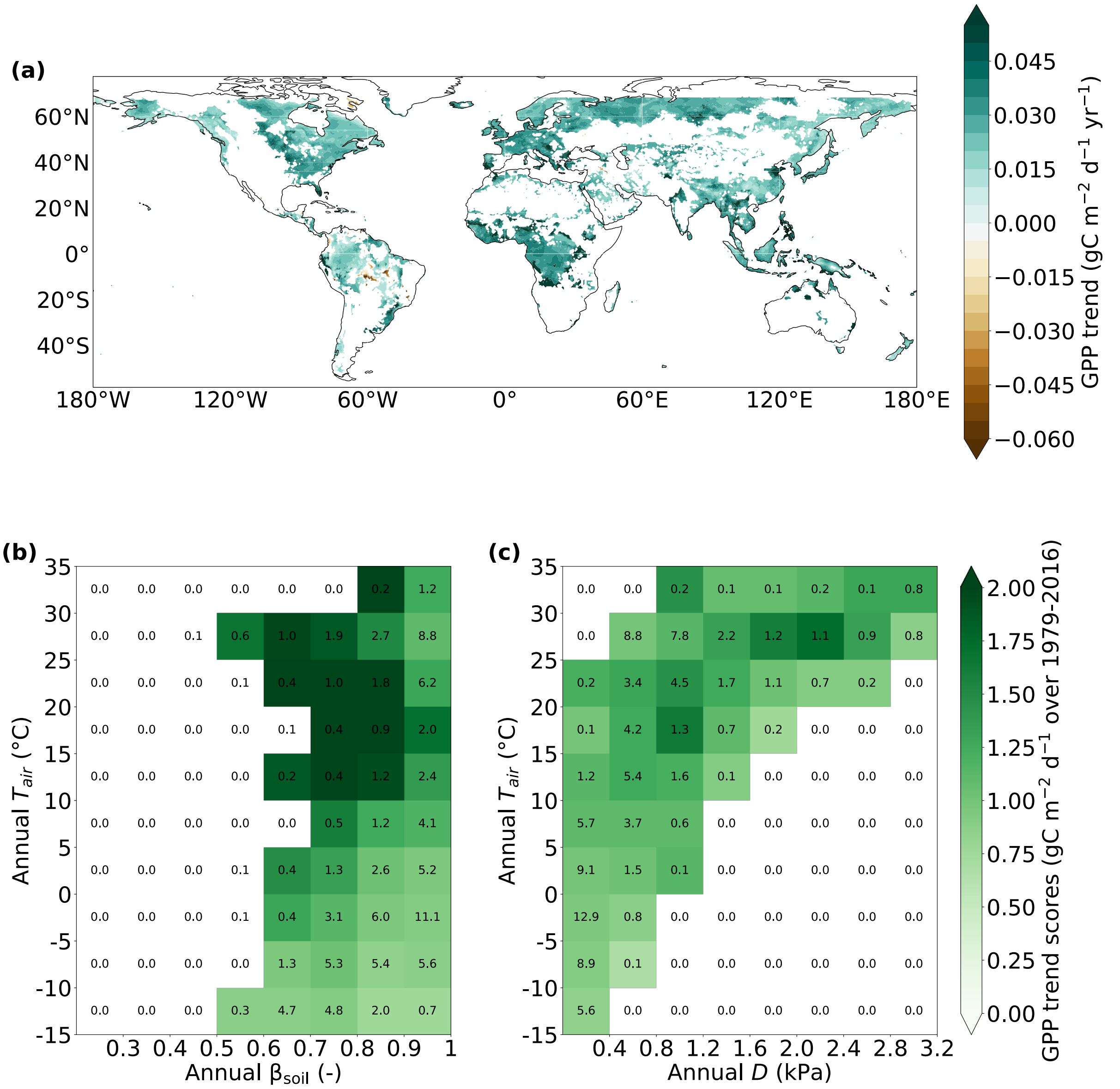

References

Farquhar, G. D., von Caemmerer, S., & Berry, J. A. (1980). A Biochemical Model of Photosynthetic CO2 Assimilation in Leaves of C3 Species. *Planta, 149*, 78-90. https://doi.org/10.1007/BF00386231

Harper, A. B., Cox, P. M., Friedlingstein, P., Wiltshire, A. J., Jones, C. D., Sitch, S., Mercado, L. M., Groenendijk, M., Robertson, E., Kattge, J., Bönisch, G., Atkin, O. K., Bahn, M., Cornelissen, J., Niinemets, Ü., Onipchenko, V., Peñuelas, J., Poorter, L., Reich, P. B., Soudzilovskaia, N. A., & Bodegom, P. v. (2016). Improved representation of plant functional types and physiology in the Joint UK Land Environment Simulator (JULES v4.2) using plant trait information. *Geoscientific Model Development, 9*(7), 2415-2440. https://doi.org/10.5194/gmd-9-2415-2016

Kattge, J., Knorr, W., Raddatz, T., & Wirth, C. (2009). Quantifying photosynthetic capacity and its relationship to leaf nitrogen content for global-scale terrestrial biosphere models. *Global Change Biology, 15*(4), 976-991. https://doi.org/10.1111/j.1365-2486.2008.01744.x

Medlyn, B. E., Badeck, F., De Pury, D. G. G., Barton, C. V. M., Broadmeadow, M. S. J., Ceulemans, R., De Angelis, P., Forstreuter, M., Jach, M. E., Kellomaki, S., Laitat, E., Marek, M., Philippot, S., Rey, A., Strassemeyer, J., Laitinen, K., Liozon, R., Portier, B., Roberntz, P., Wang, K., & Jarvis, P. G. (1999). Effects of elevated [CO2] on photosynthesis in European forest species: a meta-analysis of model parameters. *Plant, Cell & Environment, 22*, 1475-1495. https://doi.org/10.1046/j.1365-3040.1999.00523.x

Medlyn, B. E., Dreyer, E., Ellsworth, D., Forstreuter, M., Harley, P. C., Kirschbaum, M. U. F., Le Roux, E., Montpied, P., Strassemeyer, J., Walcroft, A., Wang, K., & Loustau, D. (2002). Temperature response of parameters of a biochemically based model of photosynthesis. II. A review of experimental data. *Plant, Cell & Environment, 25*, 1167-1179. https://doi.org/10.1046/j.1365-3040.2002.00891.x
